# Salt stress enhances bioactive compound accumulation in Glycyrrhiza inflata: integrated transcriptomics and physiological analysis reveals germplasm-specific adaptation mechanisms

**DOI:** 10.3389/fpls.2025.1658530

**Published:** 2025-09-03

**Authors:** Bo Zhu, LinYuan Cheng, Nana Shi, Pizheng Chen, Fei Guo, Yiyuan Qu, Hua Yao, Haitao Shen

**Affiliations:** ^1^ Key Laboratory of Xinjiang Phytomedicine Resource and Utilization, Ministry of Education, College of Life Sciences, Shihezi University, Shihezi, China; ^2^ School of Life Sciences, Zhuhai College of Science and Technology, Zhuhai, China; ^3^ Department of Industry and Information Technology of Xinjiang Uygur Autonomous Region, Urumqi, China; ^4^ Key Laboratory of Oasis Town and Mountain-Basin System Ecology of Xinjiang Production and Construction Corps, Shihezi University, Shihezi, China

**Keywords:** salt stress, *Glycyrrhiza inflata*, germplasm, bioactive compounds, transcriptomics

## Abstract

**Introduction:**

Glycyrrhiza inflata Batal., a halophytic plant predominantly found in the saline-alkali deserts of southern Xinjiang, China, is renowned for its abundance of bioactive compounds like flavonoids and triterpenoids. It demonstrates considerable potential for applications within the pharmaceutical, food, health product, and cosmetic industries. Additionally, its cultivation presents the dual advantage of generating economic returns and facilitating the remediation of saline-alkali soils.

**Methods:**

This study examined 29 distinct provenances of G. inflata collected from various locations across Xinjiang. Key agronomic traits and the content of bioactive compounds in the underground parts of one-year-old plants grown in severely saline-alkali soil were measured to assess inter-germplasm variation. Subsequently, four germplasms displaying contrasting quality and salt tolerance were selected for controlled salt stress treatment (150 mM NaCl) under laboratory conditions. The effects on seed germination, root bioactive compound content, endogenous hormone levels, and key physiological and biochemical indices were analyzed. An integrated analysis of salt stress transcriptomic data was conducted using Weighted Gene Co-expression Network Analysis (WGCNA). This involved expression clustering and enrichment analysis of differentially expressed genes (DEGs) to investigate the impact of salt stress on genes related to bioactive compound biosynthesis (particularly flavonoids), endogenous hormone pathways, and key flavonoid biosynthesis enzymes.

**Results:**

The findings indicate that germplasms with superior stress tolerance maintained higher and more stable levels of antioxidant enzymes. In response to stress, these resilient germplasms modulated hormone signaling, notably upregulating abscisic acid (ABA) and downregulating auxin (IAA), thereby reallocating resources towards defense mechanisms. Crucially, salt stress was identified as an effective means to enhance the accumulation of bioactive compounds in G. inflata. Transcriptomic analysis revealed substantial divergence in post-stress gene expression patterns among germplasms, implicating key pathways such as plant hormone signal transduction, flavonoid biosynthesis, and phenylpropanoid metabolism.

**Discussion:**

This research establishes a foundation for breeding high-quality G. inflata germplasms adapted to desert saline-alkali environments and provides valuable insights into the molecular mechanisms regulating the synthesis and accumulation of its valuable bioactive compounds.

## 1 Introduction

Soil salinization is a major contributor to soil degradation, reduced crop yields, and ecosystem imbalance. This process has significantly compromised the sustainability of agricultural production systems and jeopardizes global food security (Hassani et al., 2021) Globally, approximately one-third of arable land is located in arid and semi-arid regions. These areas are frequently affected by salinization. Soil salinization adversely impacts crops through multiple mechanisms, high salt concentrations impede seed water imbibition, suppressing germination rates; It also restricts root development, impairing water and nutrient uptake. This subsequently inhibits shoot growth, leading to stunted plants, chlorosis, wilting, and even mortality. Furthermore, salinization alters soil microbial community structure and compromises ecosystem functions, indirectly affecting crop growth and productivity (van Zelm et al., 2020)Confronted with this urgent challenge, researchers worldwide are dedicated to developing innovative remediation technologies for saline-alkali soils. Several approaches with demonstrated potential and theoretical foundations have been established. Hydraulic engineering, a conventional method (Du et al., 2023), regulates soil water-salt dynamics through optimized irrigation and drainage. Agricultural management focuses on agronomic interventions, such as deep plowing, application of soil amendments (Dai et al., 2025) and strategic crop rotation.

Biological remediation has attracted considerable research focus in recent years, with halophyte-based approaches demonstrating distinct advantages and significant potential (Duan et al., 2022), Halophytes, defined as plants capable of thriving naturally in high-salinity environments, adapt to these conditions through specialized physiological and biochemical mechanisms (Jiao et al., 2022), These include intracellular ion homeostasis and osmoregulation (Sun et al., 2021) Beyond extracting soil salts to reduce salinity levels, halophytes enhance soil physicochemical properties via root exudates. This process promotes soil aggregate formation, increases porosity, and thereby creates favorable microenvironments for establishing other plant species (Li et al., 2022)。Currently, halophytes demonstrate substantial remediation efficacy in saline-alkali soil restoration. projects (Rahman et al., 2021), positioning them as promising candidates in bioremediation. For example, Suaeda salsa ameliorates soil conditions by modulating microbial communities and upregulating carbon fixation and sulfite oxidation genes (Tang et al., 2023), Medicago sativa cultivation elevates soil nitrogen and organic matter content, modifies nutrient profiles, and restructures fungal/bacterial community composition (Qi et al., 2023).

Among halophytes, *Glycyrrhiza inflata* Batal. emerges as a high-value psammohalophyte predominantly inhabiting saline-alkaline deserts of southern Xinjiang, China. This species exhibits remarkable environmental resilience under extreme conditions. Beyond its exceptional salt tolerance enabling survival in highly saline-alkali soils, *G. inflata* accumulates medicinally significant triterpenoids and flavonoids—including glycyrrhizin, liquiritin, and licochalcone A (Jiang et al., 2020), demonstrating potent antioxidant (Liu et al., 2024), anti-inflammatory (Wu et al., 2024),and anti-cancer activities (Meng et al., 2024). Within the global pharmaceutical market, *G. inflata*-derived products hold significant market share across Asia, Europe, and North America (Ma et al., 2025a),attributed to their unique therapeutic properties. Consequently, research and utilization of this species offers a viable bioremediation strategy for decertified saline-alkali lands while simultaneously stimulating regional economic growth through industry development and job creation.

Recent trends reveal a critical conservation status of wild *Glycyrrhiza inflata* resources (Yan et al., 2023),with dwindling populations failing to meet current industrial and medicinal demands for both quantity and quality. While commercially cultivated alternatives provide partial substitution, their inconsistent quality remains a significant limitation (Wang et al., 2019),Consequently, elucidating the salt and drought tolerance mechanisms of *G. inflata* transcends academic interest—it represents an urgent research imperative. This work is fundamental to ensuring sustainable resource supply and advancing evidence-based cultivation practices for the licorice industry.

Given that glycyrrhizin and liquiritin—classified as triterpenoid and flavonoid markers respectively (Yang et al., 2017), serve as critical quality determinants for licorice, enhancing their biosynthesis presents an urgent research priority. Recent advances in plant stress physiology demonstrate that moderate salt stress effectively stimulates bioactive compound accumulation across diverse species (Wang et al., 2020; Gu et al., 2024; Li et al., 2024), This evidence-based approach offers a novel pathway for optimizing *G. inflata* phytochemical yield.

We initiated germplasm screening by conducting germination assays and quantifying root glycyrrhizin/liquiritin content in 29 *G. inflata* accessions from severely saline-alkali soils, selecting elite germplasms exhibiting superior salt tolerance, high germination rates, and enhanced phytochemical profiles. Two salt-tolerant and two salt-sensitive accessions with contrasting metabolite accumulation were then subjected to controlled salt stress (150 mM NaCl). Treatment effects on germination dynamics, root bioactive compounds, endogenous hormones, and physiological indices were analyzed. Subsequent transcriptomic investigation integrated differential gene expression profiling, co-expression clustering, and functional enrichment to elucidate salt stress impacts on bioactive compound biosynthesis, hormone signaling, and flavonoid pathway genes. Finally, Weighted Gene Co-expression Network Analysis (WGCNA) (Liu et al., 2019) identified hub genes co-regulated with stress tolerance and flavonoid accumulation, revealing preliminary mechanistic relationships between specialized metabolism and stress adaptation.

## 2 Materials and methods

### 2.1 Plant materials and treatments

All 29 *Glycyrrhiza inflata* accessions used were pure Xinjiang germplasms previously collected and authenticated by our laboratory. Seeds underwent 50-min 98% sulfuric acid treatment to break dormancy and enhance germination, followed by thorough rinsing to remove residues. Field cultivation occurred at Shihezi University Experimental Station (44°16’N, 86°00’E) under temperate continental climate (≈170-day frost-free period; July mean: 25–26°C; annual precipitation: 125–207 mm). Seeds were planted in May using drip irrigation (2.05-m wide mulch, six rows per bed, three drip lines) with alternating wide-narrow row spacing (18,000 plants/ha; irrigation: 4,200 m³/ha). Roots (20 cm segments from 5 plants/accession) were harvested during late September–early October for yield assessment (fresh/dry weight) and phytochemical analysis.

Saline-alkali field trials were conducted in Minfeng County (82°22’E, 35°20’N) featuring typical temperate desert climate (mean annual temp: stable; precipitation: 30.5 mm; evaporation: 2,756 mm; frost-free period: 194 days; sunshine: 2,842.2 hr). Identical root sampling protocols were followed.

Germination assays used plump, uniform seeds (50 seeds/dish on filter paper; 3 treatments × 3 replicates) with 10 mL test solution replenished periodically using original concentrations to maintain osmolarity (distilled water control). Salt stress experiments proceeded in climate-controlled chambers (200 μmol·m⁻²·s⁻¹ PPFD; 16/8-h light/dark; 50–55% RH; 28°C/25°C day/night). Seeds were sown in vermiculite-filled pots (10×10×10 cm; 6 plants/pot), watered biweekly and fertilized weekly with half-strength MS medium. At 20-cm height, plants received 150 mM NaCl (pre-optimized concentration). Aboveground and root tissues (harvested at root-shoot junction) were sampled at 0, 2, 6, 12, and 24 h post-treatment (three biological replicates). Samples were rinsed, flash-frozen in liquid nitrogen, and stored at −80 °C for subsequent phytochemical quantification and RNA extraction (transcriptome sequencing).

### 2.2 Determination of soil salinity and major elements

The measurement of this part was carried out by a third-party institution, the Agricultural Science Research Institute of the 7th Division of Xinjiang Production and Construction Corps. They took five samples from the 4,500 mu of land in Area 1 of the Salayuketek Township, Minfen County, Xinjiang Province, at depths of 0–30 cm and 30–60 cm. The pH measurement results of the 0–30 cm soil samples were 8.27, with water-soluble salts at 5.33 g/kg, available nitrogen at 1.62 mg/kg, available phosphorus at mg/kg, and available potassium at 106 mg/kg. The pH measurement results of the 30 –60 cm soil samples were 8.15, with water-soluble salts at 6.00 g/kg, available nitrogen at 0.82 mg/kg, available phosphorus at 0.09 mg/kg, and available potassium at 66 mg/kg.

### 2.3 Extraction and determination of active pharmaceutical components

Powdered samples (1.000 g) were ultrasonically extracted with 5 mL 100% methanol at 25°C for 60 min (1000 W), followed by centrifugation at 12,000 rpm (4°C, 5 min). Supernatants were collected, and residues were re-extracted with 5 mL methanol. Combined supernatants were diluted to volume in a volumetric flask with methanol. Aliquots (2.5 mL) were syringe-filtered through 0.22 µm membranes for phytochemical analysis. Standard solutions (1.0 mg/mL) were serially diluted in methanol to construct calibration curves (1 – 100 ng/mL). Quantitative analysis was performed using an ACQUITY UPLC H-Class system (Waters) coupled to a Xevo TQS triple quadrupole mass spectrometer. Separation was achieved on an ACQUITY UPLC BEH C18 column (50 × 2.1 mm, 1.7 μm; Waters) maintained at 40°C with a 0.3 mL/min flow rate. The mobile phase consisted of (A) 0.1% formic acid in water and (B) acetonitrile, using the following gradient: 0–2 min 5% B, 2–8 min 5-95% B, 8–10 min 95% B, 10-10.1 min 95-5% B, 10.1–12 min 5% B.

### 2.4 Extraction and determination of total flavonoids from licorice

For total flavonoid quantification, 2.00 g of licorice root powder was accurately weighed, wrapped in filter paper, and subjected to Soxhlet extraction with ethanol under 80°C water bath reflux until colorless effluent was observed. The extract was concentrated under reduced pressure, transferred to a 100-mL volumetric flask, and diluted to volume with ethanol. A 0.5-mL aliquot of this solution was mixed with 2.5 mL distilled water in a 10-mL volumetric flask. Sequentially, 0.5 mL of 5% NaNO_2_ and 0.5 mL of 10% Al(NO_3_)_3_ were added with vortex mixing after each addition. After 5-min incubation at room temperature, 2.5 mL of 5% NaOH was added and mixed thoroughly. Following additional 5-min incubation, the solution was diluted to volume with distilled water. Absorbance was measured at 520 nm against a reagent blank using UV-Vis spectrophotometer.

### 2.5 Antioxidant enzyme content determination

Fresh leaf samples (0.5 g) were homogenized in 4.5 mL ice-cold 50 mM phosphate buffer (pH 7.0 - 7.4) containing 0.1 mM EDTA and 2 mM DTT. The homogenate was centrifuged at 4,000 ×g for 50 min at 4°C, and the supernatant was retained for antioxidant enzyme assays. Content of superoxide dismutase (SOD), peroxidase (POD), catalase (CAT), free proline, and malondialdehyde (MDA) were quantified in *G. inflata* roots using commercial assay kits (Nanjing Jiancheng Bioengineering Institute, China) according to manufacturer protocols.

### 2.6 Chlorophyll content measurement

Fresh tissue samples (0.1 g) were finely chopped and transferred to 50-mL conical flasks containing 10 mL of 95% (v/v) ethanol. After 3 – 5 h of dark incubation at room temperature until complete decolorization occurred, extracts were filtered into 25-mL amber volumetric flasks. The mortar and pestle were rinsed three times with small volumes of 95% ethanol, with washings filtered into the same flask. The combined filtrate was diluted to volume with ethanol and homogenized. Extract aliquots were transferred to cuvettes against a 95% ethanol blank. Absorbance was measured at 665 nm and 649 nm using UV-Vis spectrophotometer.

### 2.7 Transcriptome analysis

Total RNA was isolated using the Polyphenolics-Polysaccharides RNA Kit (TIANGEN Biotech, China). cDNA library construction was performed by Majorbio Bio-Pharm Technology Co., Ltd. Poly(A)+ mRNA was enriched from qualified RNA samples using oligo(dT)-attached magnetic beads. Following fragmentation (~300 bp fragments compatible with Illumina HiSeq 6000 platform requirements), first-strand cDNA synthesis was conducted with reverse transcriptase. Second-strand cDNA synthesis generated stable double-stranded DNA, which underwent end repair, adapter ligation, and PCR amplification for library enrichment. Thirty-six cDNA libraries were constructed, representing four *G. inflata* accessions under salt stress across two timepoints with triplicate biological replicates. Libraries were sequenced on the Illumina HiSeq 6000 platform (150-bp paired-end). Raw reads underwent quality control using SeqPrep and Sickle (Chen et al., 2025) to obtain clean data. High-quality reads were aligned to the G. uralensis reference genome (v2.0) using TopHat2 (Kim et al., 2013)and HISAT2 (Kim et al., 2019).Gene expression quantification was performed with RSEM (Li and Dewey, 2011). Differential expression analysis employed DEGseq (Wang et al., 2010)and DESeq2 (Love et al., 2014) (|log_2_FC| > 1, FDR < 0.05). Functional annotation utilized DIAMOND for GO terms and KEGG Orthology (KO) assignment. Enrichment analyses were conducted with GOATools (Klopfenstein et al., 2018)and KOBAS (Bu et al., 2021) (FDR < 0.05). Weighted Gene Co-expression Network Analysis (WGCNA) (Liu et al., 2019)was implemented in R v4.2.2 to identify salt-responsive genes associated with specialized metabolism. An adjacency matrix was constructed using soft thresholding (power = β = 14). Modules were detected via dynamic tree cutting (mergeCutHeight = 0.25, minModuleSize = 30). Module eigengenes (first principal components) represented expression profiles. Spearman correlations between module eigengenes and flavonoid abundances were visualized using ggplot2.

### 2.8 Real-time fluorescence quantitative PCR

To validate RNA-seq accuracy, six differentially expressed genes (DEGs) regulating flavonoid biosynthesis were selected for qRT-PCR verification(**Supplementary Figure S3**). Specific primers were designed to measure relative expression levels of these genes in root tissues of four *G. inflata* accessions after 6-h salt treatment. The Glycyrrhiza uralensis lectin gene (Glyur000100s00008376) served as the endogenous reference. Reactions utilized the FastStart Universal SYBR Green Master Mix (Roche) on an Applied Biosystems 7500 Real-Time PCR System (Monad Biotech, China). Relative transcript abundance was calculated via the 2^–ΔΔCt^ method (Livak and Schmittgen, 2001), Statistical significance was determined by one-way ANOVA with Tukey’s *post-hoc* test (p < 0.05).

### 2.9 Statistical analysis

All statistical analyses were performed using R packages and SPSS 27.0. Intergroup differences were assessed by one-way analysis of variance (ANOVA), with *post-hoc* pairwise comparisons conducted via the least significant difference (LSD) test at a significance threshold of P < 0.05.

## 3 Result

### 3.1 Quantification of bioactive compounds and saline-alkali germination rates in one-year-old cultivated *G. inflata*

**Figure 1:**
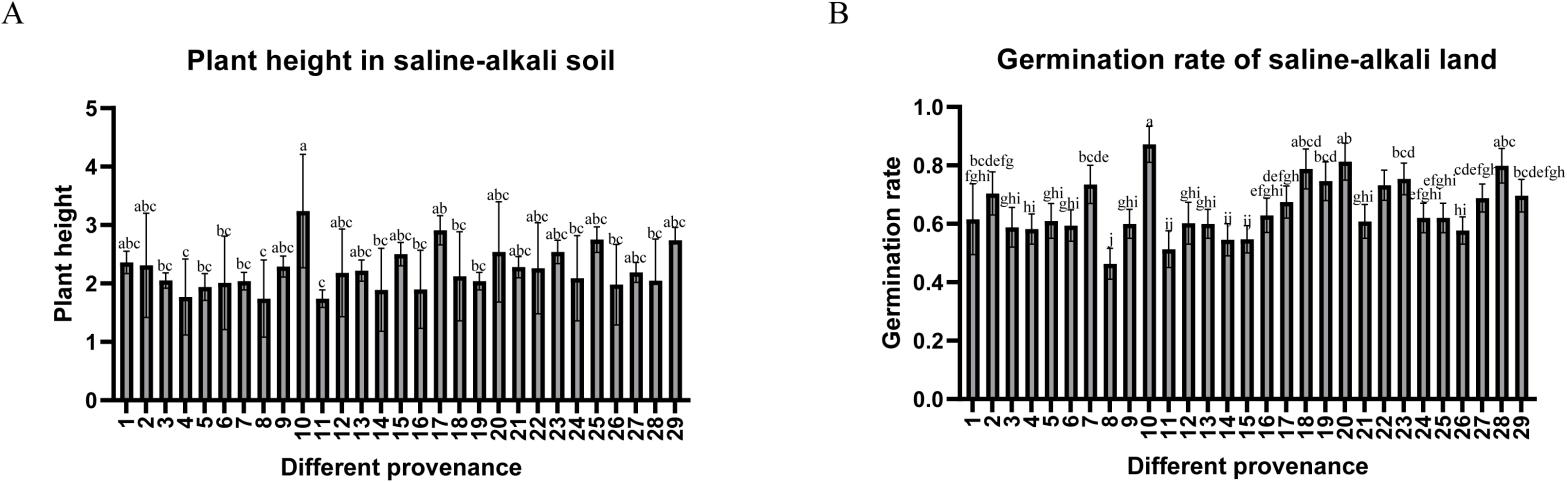
**(A)** The average plant height of 29 types of *Glycyrrhiza inflata* in the saline-alkali land experimental field. **(B)** The average germination rate of 29 types of *Glycyrrhiza inflata* in the saline-alkali land experimental field. The differences between samples were determined by one-way analysis of variance (ANOVA), and the significance difference when P < 0.05 was calculated by the least significant difference (LSD) test.

**Figure 2:**
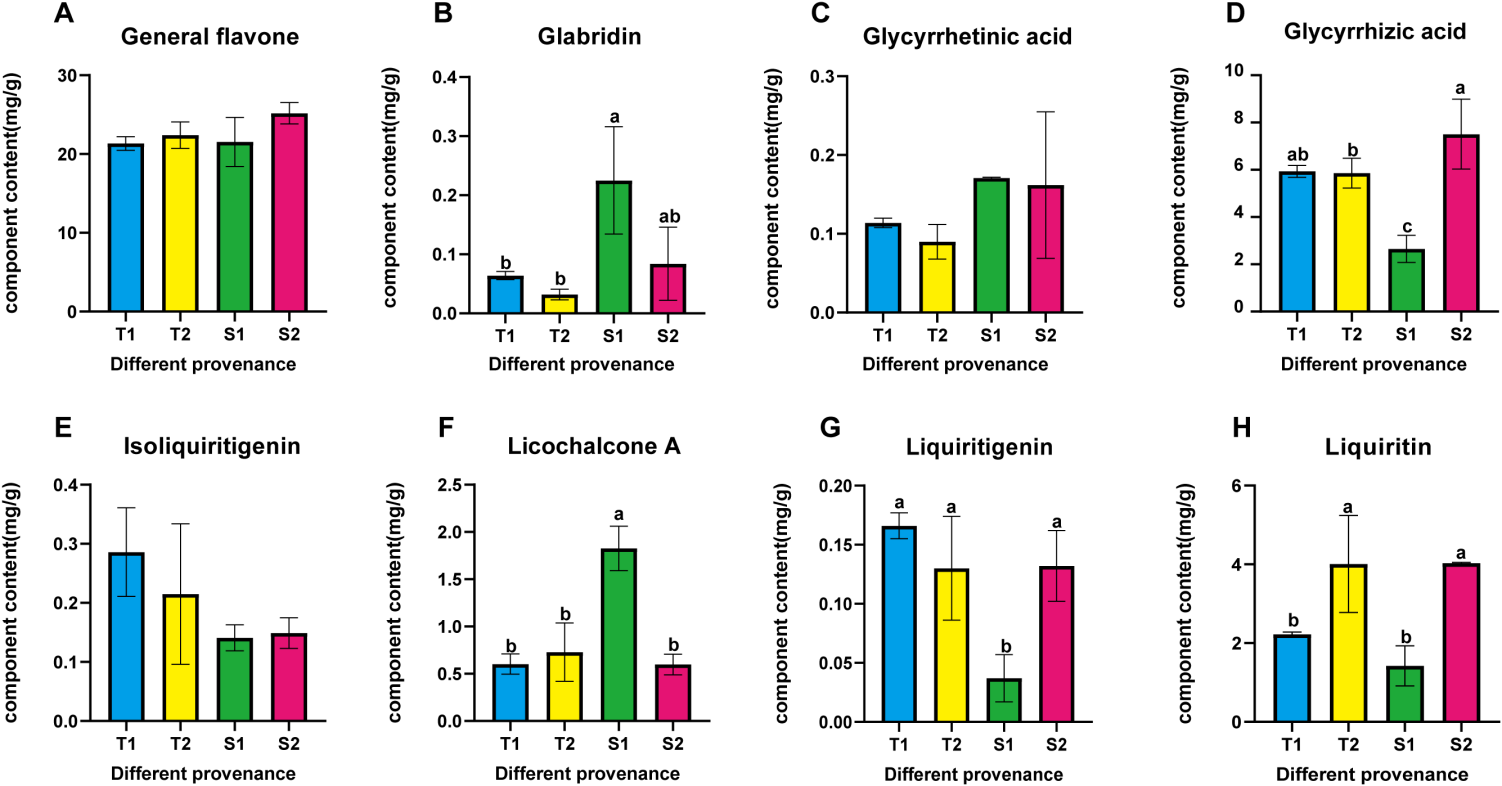
**(A–H)** respectively represent the contents of general flavonoids, glycyrrhizin, glycyrrhetinic acid, glycyrrhizic acid, isoglycyrrhizin, glycyrrhizin chalcone A, glycyrrhizin and glycyrrhizin in four different provenance *Glycyrrhiza inflata*. All Data represent root tissue samples only. The differences between samples were determined by one-way analysis of variance (ANOVA), and the significance difference when P < 0.05 was calculated by the least significant difference (LSD) test.

Confronting the dual challenges of diminishing wild *Glycyrrhiza inflat*a resources and declining cultivated quality, we evaluated 29 germplasms from the Tarim Basin periphery (coordinates in **Supplementary Table S1**), revealing significant inter-accession variation in bioactive compounds (**Table 1**) and salt tolerance, as shown in **Figure 1**,all accessions achieved >45% germination in saline-alkali soil (pH 8.15; mean 65.13% ± 8.24%), with 20 exceeding 60% (nine >70%, two >80%, #10 peaking at 87.1%), while plant heights ranged 1.74 – 3.24 m (mean 2.23 m). Critical metabolites varied substantially: total flavonoids (17.93 ± 2.81 to 28.97 ± 5.53 mg/g), glycyrrhizic acid (2.06 ± 0.23 to 7.51 ± 1.48 mg/g), liquiritin (0.72 ± 0.14 to 4.01 ± 1.23 mg/g), liquiritigenin (0.05 ± 0.01 to 0.22 ± 0.03 mg/g), and isoliquiritigenin (0.09 ± 0.01 to 0.29 ± 0.01 mg/g), with hyper-arid desert margin accessions (#1,9,15) showing superior accumulation (P<0.05), suggesting strategic salt stress enhances medicinal biosynthesis. Through triaxial selection ranking germplasms by glycyrrhizic acid content, liquiritin content, and germination rate (**Supplementary Figure S1**)—driven by dual-purpose application in remediating southern Xinjiang’s saline-alkali deserts while generating local economic returns via harvestable bioactive compounds—we prioritized accessions meeting critical germination thresholds due to severely compromised viability in extreme salinity-alkalinity soils, thus selecting: stress-tolerant accessions (#10=T1, #28=T2) with high germination and enriched bioactive compounds, and sensitive accessions (#22=S1, #13=S2) exhibiting moderate germination rates (relatively lower than T1/T2) yet divergent bioactive compound accumulation for comparative stress-response analysis. their controlled-environment metabolite profiles (**Figure 2**) indicated no significant differences in general flavonoids, or isoglycyrrhizin (P>0.05), but glabridin, licochalcone A,liquiritigenin, liquiritin, and glycyrrhizic acid, The contents of these five active ingredients have varied.

**Table 1:** The content of medicinal active components of 29 kinds of Glycyrrhiza inflata.

Seed number	General flavonoid(mg/g)	Glycyrrhizic acid (mg/g)	Glycyrrhizin(mg/g)	Glycyrrhizin(mg/g)	Isoglycyrrhizin(mg/g)	Glycyrrhetinic acid (mg/g)	Glabridin (mg/g)	Glycyrrhetinic(mg/g)
1	26.523 ± 1.210 abcd	7.615 ± 0.185 ab	3.656 ± 0.155 ab	0.133 ± 0.016 cd	0.287 ± 0.011 a	0.943 ± 0.015 fghij	0.125 ± 0.014 ef	0.227 ± 0.010 abc
2	20.381 ± 0.351 defg	4.115 ± 0.809 bcde	1.902 ± 0.359 def	0.104 ± 0.001 defg	0.201 ± 0.055 bcdef	1.109 ± 0.219 defghij	0.242 ± 0.065 bcdef	0.170 ± 0.014 bcdef
3	28.966 ± 5.533 a	3.599 ± 0.258 bcde	1.752 ± 0.077 def	0.061 ± 0.004 ghijkl	0.195 ± 0.043 bcdefg	3.071 ± 0.720 a	0.372 ± 0.219 abcd	0.187 ± 0.064 abcde
4	20.426 ± 1.995 defg	3.099 ± 0.533 de	1.593 ± 0.252 def	0.051 ± 0.006 ijkl	0.157 ± 0.005 cdefgh	1.312 ± 0.001 cdefghi	0.060 ± 0.009 f	0.126 ± 0.049 defgh
5	24.097 ± 5.492 abcdefg	2.953 ± 0.983 de	2.621 ± 0.363 bcde	0.073 ± 0.032 ghijkl	0.189 ± 0.045 bcdefg	1.139 ± 0.125 defghij	0.327 ± 0.192 abcde	0.138 ± 0.005 defg
6	18.968 ± 1.041 efg	2.515 ± 0.804 e	1.191 ± 0.016 ef	0.041 ± 0.013 jkl	0.148 ± 0.014 defgh	1.156 ± 0.424 defghij	0.135 ± 0.091 def	0.140 ± 0.115 defg
7	22.821 ± 2.329 abcdefg	4.723 ± 1.416 bcde	1.696 ± 0.836 def	0.215 ± 0.029 a	0.231 ± 0.014 abc	1.300 ± 0.362 cdefghij	0.148 ± 0.061 def	0.192 ± 0.007 abcd
8	18.511 ± 1.722 fg	2.323 ± 0.135 e	1.110 ± 0.003 ef	0.068 ± 0.012 ghijkl	0.144 ± 0.009 efgh	1.422 ± 0.157 bcdefgh	0.235 ± 0.171 cdef	0.102 ± 0.001 fgh
9	25.171 ± 1.363 abcde	7.506 ± 1.480 abc	4.029 ± 0.020 ab	0.132 ± 0.030 cd	0.165 ± 0.038 bcdefgh	0.598 ± 0.109 ij	0.084 ± 0.062 ef	0.162 ± 0.093 cdef
10	21.331 ± 0.860 cdefg	5.935 ± 0.256 abcde	2.222 ± 0.061 cdef	0.166 ± 0.011 bc	0.286 ± 0.075 a	0.602 ± 0.107 ij	0.064 ± 0.007 f	0.114 ± 0.006 defgh
11	26.175 ± 0.372 abcd	5.155 ± 0.293 abcde	1.699 ± 0.125 def	0.091 ± 0.024 efghk	0.225 ± 0.016 abcde	1.912 ± 0.150 bc	0.475 ± 0.099 ab	0.231 ± 0.037 abc
12	18.488 ± 6.388 fg	3.367 ± 1.499 de	2.186 ± 1.015 cdef	0.069 ± 0.008 ghijkl	0.142 ± 0.005 fgh	0.735 ± 0.713 hij	0.062 ± 0.039 f	0.093 ± 0.036 fgh
13	22.971 ± 2.944 abcdefg	2.489 ± 0.899 e	1.271 ± 0.740 def	0.033 ± 0.012 l	0.113 ± 0.028 gh	1.522 ± 0.727 bcdefg	0.258 ± 0.301 bcdef	0.129 ± 0.055 defgh
14	23.684 ± 1.669 abcdefg	3.790 ± 1.340 bcde	1.666 ± 1.056 def	0.096 ± 0.056 defgh	0.228 ± 0.094 abcd	1.791 ± 0.761 bcd	0.528 ± 0.142 a	0.169 ± 0.085 bcdef
15	27.415 ± 1.474 abc	8.886 ± 0.449 a	3.724 ± 0.110 ab	0.118 ± 0.036 def	0.115 ± 0.019 gh	0.776 ± 0.033 ghij	0.075 ± 0.018 f	0.254 ± 0.016 a
16	17.926 ± 2.810 g	2.056 ± 0.231 e	0.717 ± 0.139 f	0.079 ± 0.013 fghijk	0.182 ± 0.006 bcdefg	2.125 ± 0.395 b	0.251 ± 0.121 bcdef	0.112 ± 0.031 defgh
17	19.935 ± 0.326 defg	3.214 ± 0.061 de	1.322 ± 0.049 def	0.050 ± 0.009 ijkl	0.135 ± 0.017 fgh	1.328 ± 0.212 cdefghi	0.241 ± 0.020 bcdef	0.129 ± 0.023 defgh
18	22.096 ± 1.223 bcdefg	4.330 ± 0.763 bcde	2.169 ± 0.262 cdef	0.075 ± 0.022 ghijkl	0.161 ± 0.017 bcdefgh	1.036 ± 0.013 efghij	0.054 ± 0.006 f	0.054 ± 0.006 h
19	28.590 ± 1.937 ab	7.068 ± 3.021 abcd	2.747 ± 0.398 bcd	0.083 ± 0.023 fghij	0.212 ± 0.066 abcdef	1.539 ± 0.414 bcdef	0.096 ± 0.017 ef	0.104 ± 0.060 efgh
20	24.907 ± 8.714 abcdef	4.809 ± 1.169 bcde	3.439 ± 1.759 abc	0.067 ± 0.017 ghijkl	0.180 ± 0.028 bcdefg	1.332 ± 0.422 cdefghi	0.107 ± 0.086 ef	0.077 ± 0.011 gh
21	23.965 ± 1.649 abcdefg	3.525 ± 0.594 bcde	1.809 ± 0.066 def	0.062 ± 0.004 ghijkl	0.160 ± 0.019 cdefgh	1.532 ± 0.104 bcdef	0.167 ± 0.155 def	0.087 ± 0.004 fgh
22	21.525 ± 3.108 cdefg	2.655 ± 0.572 e	1.422 ± 0.512 def	0.037 ± 0.020 kl	0.141 ± 0.022 fgh	1.826 ± 0.236 bcd	0.225 ± 0.091 cdef	0.171 ± 0.001 bcdef
23	24.294 ± 5.953 abcdefg	5.599 ± 1.999 abcde	4.231 ± 2.732 a	0.133 ± 0.021 cd	0.174 ± 0.049 bcdefg	0.559 ± 0.279 j	0.044 ± 0.010 f	0.144 ± 0.044 defg
24	22.081 ± 1.212 bcdefg	3.391 ± 0.049 cde	1.411 ± 0.066 def	0.078 ± 0.012 fghijk	0.172 ± 0.021 bcdefg	1.169 ± 0.095 cdefghij	0.354 ± 0.439 abc	0.159 ± 0.025 cdefg
25	22.965 ± 0.052 abcdefg	4.709 ± 0.856 bcde	1.802 ± 0.102 def	0.188 ± 0.024 ab	0.242 ± 0.018 ab	1.200 ± 0.149 cdefghij	0.442 ± 0.017 abc	0.104 ± 0.014 efgh
26	22.985 ± 2.476 abcdefg	4.235 ± 0.127 bcde	2.170 ± 0.114 cdef	0.059 ± 0.012 hijkl	0.142 ± 0.026 fgh	1.411 ± 0.109 bcdefgh	0.058 ± 0.008 f	0.091 ± 0.008 fgh
27	19.891 ± 0.428 defg	3.652 ± 0.189 bcde	1.627 ± 0.044 def	0.060 ± 0.011 hijkl	0.158 ± 0.009 cdefgh	1.733 ± 0.171 bcde	0.164 ± 0.014 def	0.133 ± 0.033 defgh
28	22.388 ± 1.680 bcdefg	5.860 ± 0.627 abcde	4.008 ± 1.232 ab	0.130 ± 0.044 cde	0.215 ± 0.119 abcdef	0.728 ± 0.308 hij	0.032 ± 0.009 f	0.090 ± 0.022 fgh
29	23.201 ± 1.084 abcdefg	5.813 ± 0.009 abcde	3.501 ± 0.362 abc	0.067 ± 0.003 ghijkl	0.090 ± 0.001 h	1.089 ± 0.828 defghij	0.068 ± 0.012 f	0.249 ± 0.015 ab

The table above presents the measurement results of active ingredients in the roots of 29 distinct provenances of *Glycyrrhiza inflata* cultivated for one year in saline-alkali experimental fields. The differences between samples were determined by one-way analysis of variance (ANOVA), and the significance difference when P < 0.05 was calculated by the least significant difference (LSD) test.

The lowercase letters indicate significant differences among the 29 different parallel groups.

### 3.2 Physiological and biochemical responses of four *G. inflata* germplasms to salt stress

**Figure 3:**
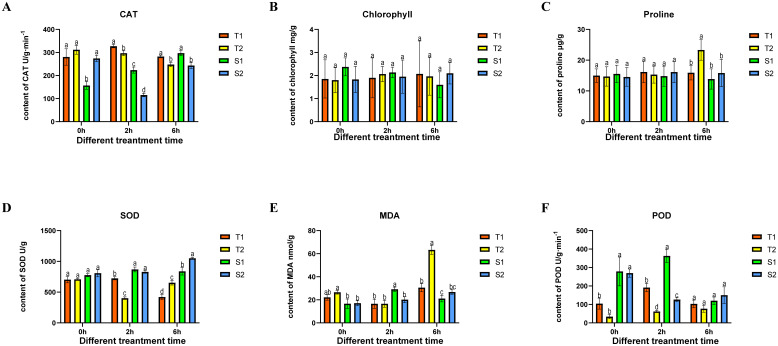
The changes in four type *Glycyrrhiza inflata* key physiological indicators after being subjected to different time periods of coercion CAT (catalase), SOD (Superoxide Dismutase), MDA (Malondialdehyde), POD (Peroxidase). The differences between samples were determined by one-way analysis of variance (ANOVA), and the significance difference when P < 0.05 was calculated by the least significant difference (LSD) test. All data represent root tissue samples only.

To elucidate salt adaptation mechanisms and select elite germplasms with enhanced stress resilience and bioactive compound production, we first assessed germination rates under varying NaCl concentrations (**Supplementary Figure S2**). After 15-day exposure to 2% NaCl, significant inter-germplasm divergence emerged: T1 (62%) > T2 (32%) > S2 (8%) > S1 (2%). When plants reached a height of 20 cm, salt stress was applied using a concentration of 150 mM NaCl, previously determined as optimal in preliminary laboratory screening. Root tissues (sampled below the root-shoot junction) were collected at 0, 2, and 6 hours after stress initiation. All samples were immediately washed with distilled water, surface-dried, flash-frozen in liquid nitrogen, and stored at -80°C, with three biological replicates per treatment. As shown in **Figure 3**, the control groups of the four Glycyrrhiza inflata varieties exhibited no significant differences in malondialdehyde (MDA), proline, or superoxide dismutase (SOD) levels. However, following salt stress, the tolerant varieties (T1, T2) accumulated significantly higher proline levels (**Figure 3C**) than the sensitive varieties (S1, S2). Conversely, MDA content (**Figure 3E**) was significantly lower in T1 and T2 compared to S1 and S2. Catalase (CAT) activity (**Figure 3A**) was consistently higher in T1 and T2 both before and after stress exposure. These differences likely contribute to the enhanced stress tolerance of T1 and T2.While chlorophyll content (**Figure 3B**) differed between tolerant and sensitive varieties, the absolute difference was relatively small compared to the other parameters (CAT, proline, SOD, MDA) and did not reach statistical significance. Interestingly, SOD activity (**Figure 3D**) in the sensitive varieties (S1, S2) was significantly higher than in the tolerant varieties (T1, T2) after stress and increased progressively with prolonged stress duration. This result contradicts our initial expectations, and the underlying mechanism remains unclear, representing a key question for future investigation.

### 3.3 Phytohormonal dynamics in four *G. inflata* germplasms under salt stress

**Figure 4:**
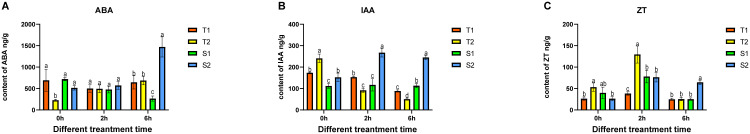
After being subjected to different time periods of stress, the changes in *Glycyrrhiza inflata* ‘s endogenous hormones. ABA (Abscisic acid), IAA (Indole-3-acetic acid), ZT (Zeatin). The differences between samples were determined by one-way analysis of variance (ANOVA), and the significance difference when P < 0.05 was calculated by the least significant difference (LSD) test.

Overall, the four Glycyrrhiza inflata varieties exhibited distinct baseline levels of the measured compounds even under non-stressed conditions, and employed divergent strategies in response to salt stress. As a key hormone in plant stress responses, abscisic acid (ABA) levels were highest in the tolerant variety T1 under control conditions and remained relatively stable after stress onset. In contrast, the other tolerant variety (T2) significantly upregulated ABA synthesis following stress exposure. Interestingly, the sensitive variety S2 also adopted this strategy of post-stress ABA induction. Conversely, ABA content in the sensitive variety S1 steadily declined after stress imposition (**Figure 4A**), an unexpected pattern that may be a key factor contributing to its stress sensitivity. Regarding indole-3-acetic acid (IAA), post-stress dynamics differed markedly between tolerant and sensitive varieties (**Figure 4B**). The tolerant varieties T1 and T2 exhibited a progressive downregulation of IAA content with prolonged stress duration. Conversely, the sensitive varieties S1 and S2 displayed an increase in IAA levels over the same period. In contrast to both ABA and IAA, the four varieties displayed a consistent pattern for zeatin (ZT) synthesis (**Figure 4C**). All varieties initially increased ZT levels shortly after stress initiation, followed by a subsequent decline as stress duration extended.

### 3.4 Salt stress modulates bioactive compound accumulation in four *G. inflata* germplasms

**Figure 5:**
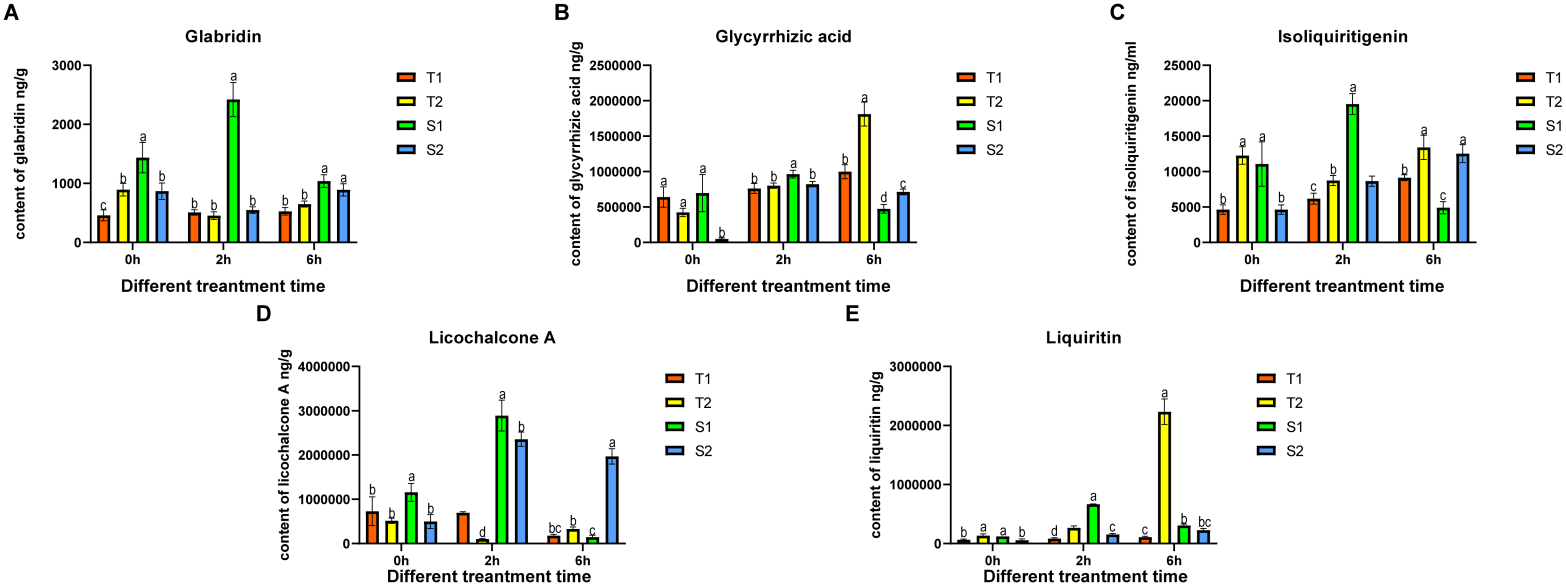
After being subjected to different time periods of stress, the content of the main active component of *Glycyrrhiza inflata* and all data represent root tissue samples only. The differences between samples were determined by one-way analysis of variance (ANOVA), and the significance difference when P < 0.05 was calculated by the least significant difference (LSD) test.

**Figure 6:**
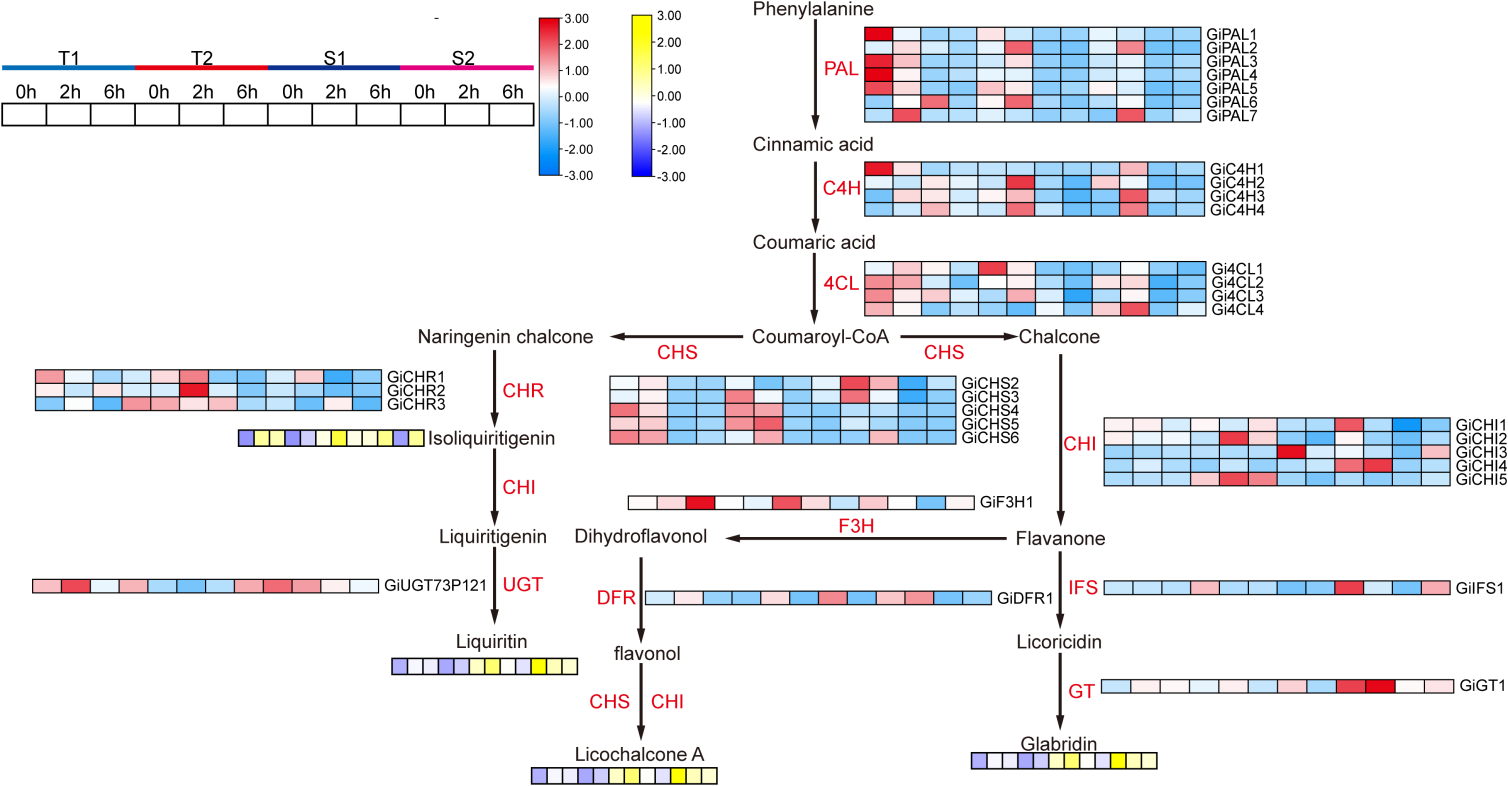
The synthesis pathway of flavonoid active components and the expression status of related genes as well as the components changes. PAL, Phenylalanine ammonia-lyase; C4H, Cinnamate 4-hydroxylase; 4CL, 4-Coumarate, Coenzyme A ligase; CHS, Chalcone synthase; CHI, Chalcone isomerase; Chalcone isomerase, Isoflavone synthase; F3H, Dihydroflavonol 3-hydroxylase; DFR, Dihydroflavonol reductase; GT, Glycosyltransferase; UGT, UDP-glucose glycosyltransferase.

Overall, short-term salt stress significantly increased the content of major active compounds in all four *Glycyrrhiza inflata* varieties, although accumulation patterns differed substantially among varieties for the same compound (**Figure 5**).As shown in **Figures 5A, D**, the sensitive varieties (S1, S2) accumulated significantly higher levels of glabridin and licochalcone A at 0, 2, and 6 hours post-stress compared to the tolerant varieties (T1, T2). Conversely, the tolerant varieties (T1, T2) exhibited greater accumulation of glycyrrhizic acid and liquiritin following stress exposure **Figures 5B, E**). Notably, glycyrrhizic acid and liquiritin are two primary compounds used in quality assessment of G. inflata. For isoliquiritigenin **Figure 5C**), accumulation patterns were similar between T1 and S2, and between T2 and S1.Analysis of the biosynthetic pathways (**Figure 6**) indicates that, with the exception of glycyrrhizic acid, the other four active compounds (glabridin, licochalcone A, liquiritin, isoliquiritigenin) are synthesized through multi-step reactions originating from coumaroyl-CoA. Notably, isoliquiritigenin and liquiritin share a common pathway, while glabridin and licochalcone A utilize the same precursor. This shared biosynthetic origin likely explains the observed similarities in their accumulation trends. The distinct accumulation patterns of these active compounds between sensitive and tolerant varieties may contribute to their differential stress tolerance.

### 3.5 Differential gene expression profiling

To validate our hypothesis and identify key salt-responsive genes in flavonoid biosynthesis, we performed transcriptome sequencing on root tissues of two salt-tolerant (T1, T2) and two salt-sensitive (S1, S2) *G. inflata* germplasms treated with 150 mM NaCl for 0, 2, and 6 hours. Differential expression analysis compared post-stress (2h/6h) versus control (0h) samples across accessions (T1_2h/T1_0h, T1_6h/T1_0h, T2_2h/T2_0h, T2_6h/T2_0h, S1_2h/S1_0h, S1_6h/S1_0h, S2_2h/S2_0h, S2_6h/S2_0h). Bar plots and Venn diagrams visualized DEG counts and overlaps (**Figures 5A, B**). At 2h post-stress, all accessions showed upregulated DEGs, but by 6h, tolerant (T1/T2) and moderately sensitive (S2) lines exhibited ∼50% reduction in upregulated DEGs (**Figure 5C**). Strikingly, S1 maintained elevated DEG counts, suggesting impaired transcriptional regulation under prolonged stress. Although total DEGs ranged 3,161-5,681 per accession, only 26 genes (0.19% of total DEGs) were commonly deregulated across all four germplasms (**Figure 5D**), indicating germplasm-specific transcriptional reprogramming.

### 3.6 Gene ontology analysis of DEGs

**Figure 7:**
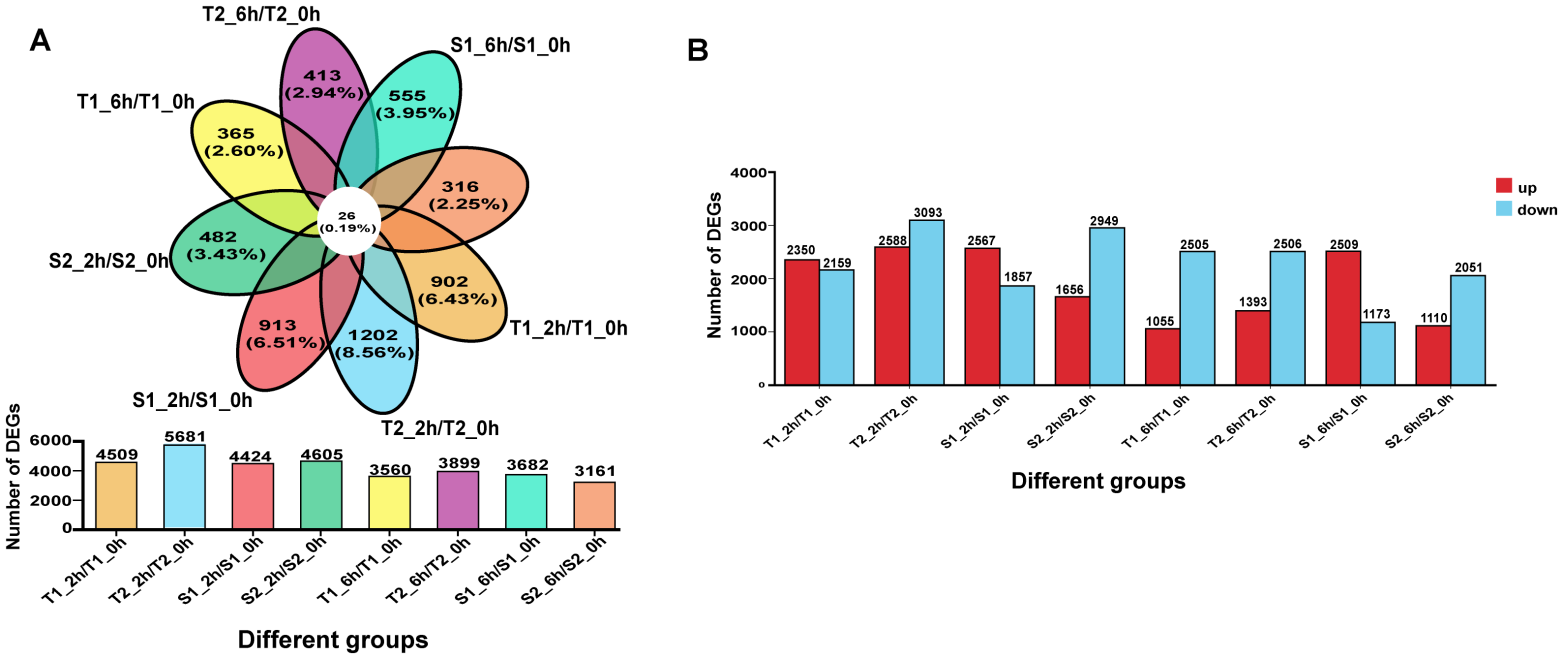
**(A)** The differences among different transcripts/overlapping gene situations. **(B)** The number of genes with upregulated/downregulated expression among different transcripts.

GO enrichment analysis of 2h/0h comparisons revealed significant enrichment (FDR < 0.05) in:Oxidative stress response, Flavonoid biosynthesis (T1:16 genes; T2:19; S1:14; S2:16),Flavonoid metabolism, Naringenin 2-hydroxylase activity, Flavonoid 3’-monooxygenase activity (exclusive to T1/S1 with 4–5 genes),Oxidoreductase activity, Hormone-mediated signaling (T1:122; T2:131; S1:91; S2:108 genes).At 6h/0h, pathways shifted toward: Isoflavonoid metabolism (S1-exclusive: 3 genes), Isoflavonoid biosynthesis, Flavonoid biosynthesis (T1:10; T2:15; S1:11; S2:10 genes),Glucosidase activity, Flavonoid 3’-monooxygenase activity (S1-exclusive: 5 genes),Hormone signaling (T1:99; S1:108; S2:81 genes; absent in T2).This temporal progression indicates transcriptional activation peaked at 2h post-stress, with flavonoid pathway genes and Hormone-mediated signaling showing maximal induction (**Figure 7E**). By 6h, attenuated activation coincided with specialized isoflavonoid pathway engagement, particularly in S1.

### 3.7 KEGG pathway enrichment of DEGs

To functionally characterize differentially expressed genes (DEGs), we performed KEGG enrichment analysis on 2h/0h and 6h/0h comparisons. The top 20 significantly enriched pathways (FDR < 0.01) shared across treatment groups revealed. At 2h post-stress:Isoflavonoid biosynthesis (ko00943), Phenylalanine metabolism (ko00360),Plant MAPK signaling (ko04016), Nitrogen metabolism (ko00910), Ubiquinone/terpenoid-quinone biosynthesis (ko00130), Phenylpropanoid biosynthesis (ko00940). Notably enriched flavonoid pathways: Flavone and flavonol biosynthesis (ko00944), Flavonoid biosynthesis (ko00941),Isoflavonoid biosynthesis (ko00943), Phenylpropanoid biosynthesis (ko00940). Concurrent enrichment of Plant hormone signal transduction (ko04075) suggests coordinated regulation. The significant enrichment (P <0.04) of multiple flavonoid biosynthesis pathways correlates with observed phytochemical upregulation (Section 3.4), indicating transcriptional reprogramming drives stress-induced metabolite accumulation.

### 3.8 Weighted gene co-expression network analysis

**Figure 8:**
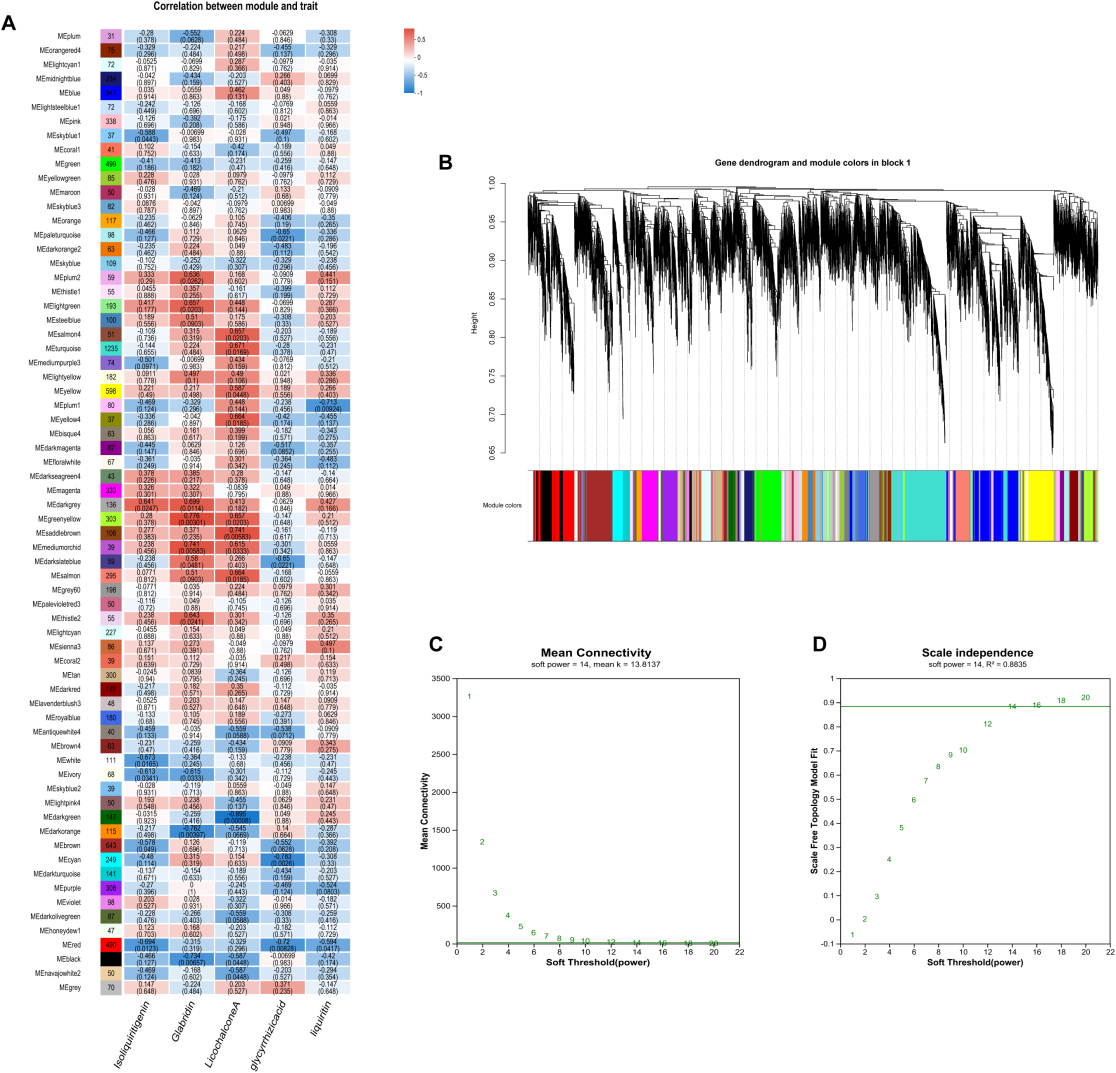
Results of the gene co-expression network analysis. **(A)** The heatmap of correlation coefficient between flavonoids contents and module eigengenes with the red and blue blocks representing positive and negative correlations, respectively; **(B)** Dendrogram showing modules identified by the weighted gene coexpression network analysis (WGCNA) and clustering dendrogram of expressed genes; **(C)** Average connectivity curve; **(D)** Scale-free wide comfort curve.

**Figure 9:**
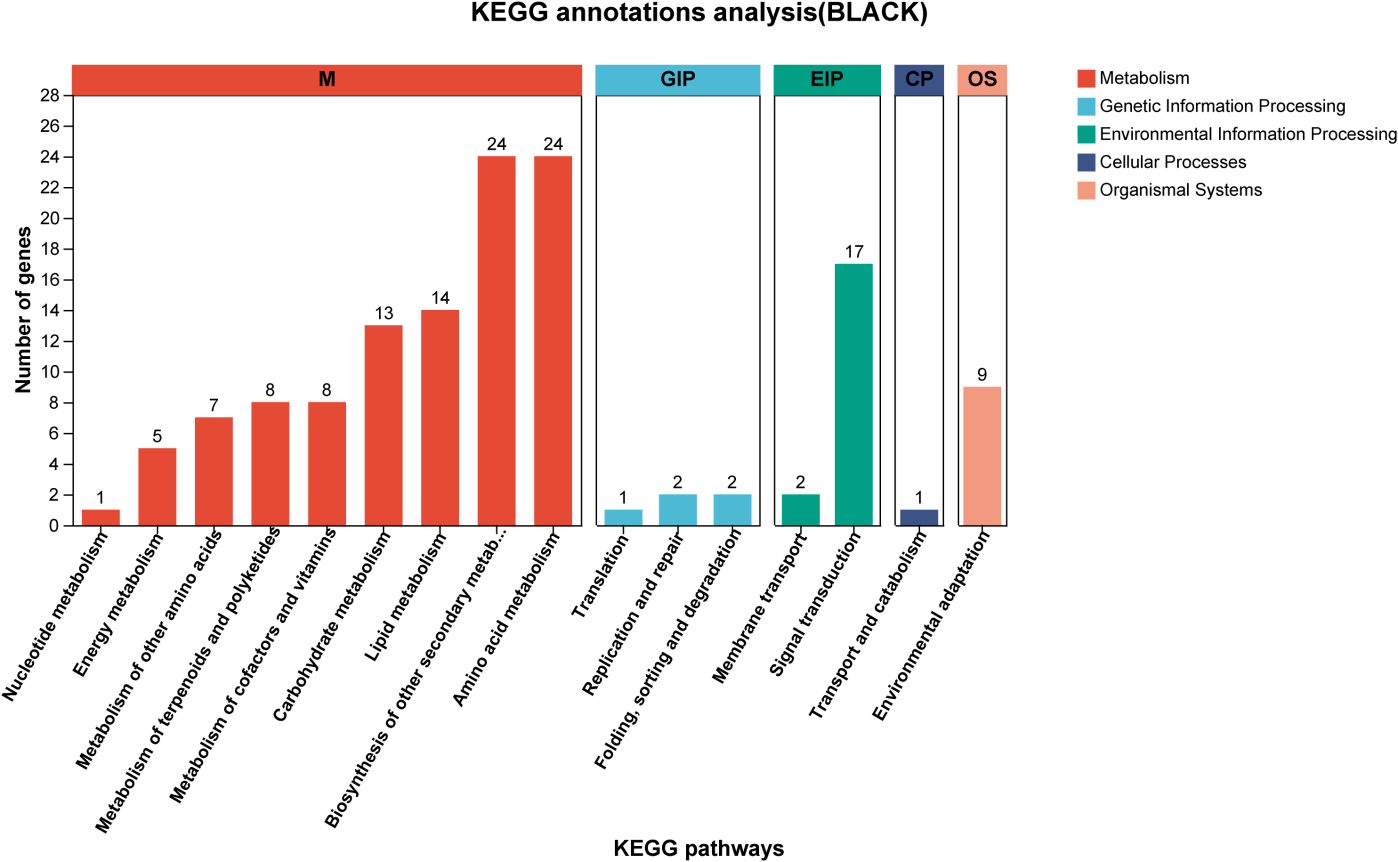
The KEGG annotation results of black module, different colors represent different functional classifications.

Using correlation-based clustering, we grouped co-expressed genes into modules via hierarchical clustering dendrograms, where branch colors represent distinct expression patterns (**Figure 8A**). WGCNA partitioned *G. inflata* genes into 68 co-expression modules, with the turquoise module being largest (1,235 genes) and plum smallest (31 genes). Module-trait associations were visualized through:Gene-trait correlation heatmap (**Figure 8B**),Mean connectivity distribution (**Figure 8C**), Scale-free topology fit index (**Figure 8D**). Spearman correlation analysis revealed significant compound-module associations: The greenyellow and black modules have the strongest correlation with Glabridin, the darkgreen module has the strongest correlation with Licochalcone A, the cyan module has the strongest correlation with glycyrrhizic acid module, and the plum1 module has the strongest correlation with Liquiritin. To further understand the relationship between the relevant modules and active components, and to identify key genes, we conducted KEGG annotation analysis on all modules with a coefficient of > 0.7. Genes encoding enzymes for primary bioactive compound synthesis were identified across multiple co-expression modules (darkorange, darkgreen, cyan), including Phenylalanine ammonia-lyase (*PAL*, *GinfChr2G00031740*) and Chalcone isomerase (*CHI*, *GinfChr1G00083500*; *GinfChr1G00083530*). Within the black module (138 genes), we detected:Five *PAL* genes (*GinfChr1G00091980*, *GinfChr2G00031750*, *GinfChr2G00031760*, *GinfChr5G00197500*, *GinfChr1G00120940*)/One Chalcone reductase (*CHR*, *GinfChr5G00197280*),One 3-Hydroxy-3-methylglutaryl-CoA reductase* (*HMGR*, *GinfChr5G00173040*),One Cinnamate 4-hydroxylase* (*C4H*, *GinfChr1G00085290*), Two 4-Coumarate: CoA ligases* (*4CL*, *GinfChr4G00082360*, *GinfChr5G00165360*), Four Chalcone synthases (*CHS*, *GinfChr3G00137770*, *GinfChr4G00071830*,*GinfChr4G00071850*).KEGG annotation revealed significant enrichment (P<0.001) in five functional categories (**Figure 9**): Metabolism (104 genes), Genetic Information Processing, Environmental Information Processing, Cellular Processes, and Organismal Systems. Metabolic genes predominated, with 24 genes each in secondary metabolite biosynthesis and amino acid metabolism. Crucially, this module contained:Phloridzin synthase (flavonoid pathway),2-Hydroxyisoflavanone dehydratase* (*HIDH*),2,7,4’-Trihydroxyisoflavanone 4’-O-methyltransferase* (*HI4OMT*),cis-Zeatin O-glucosyltransferase (cis-ZOG, EC:2.4.1.-). These findings indicate coordinated regulation of flavonoid diversification and phytohormone signaling (**Table 2**). We propose that salt stress induces crosstalk between hormone pathways (zeatin, gibberellins, ethylene, jasmonate) and specialized metabolism, particularly glabridin biosynthesis. Competitive flux partitioning may occur, where synthesis of certain flavonoids/isoflavonoids potentially downregulates glabridin-associated genes.

**Table 2:** The KEGG annotation results of black module.

Gene id	KO id	KO name	Pathway id	Category
GinfChr2G00024370.1	K14515	EBF1_2	map04075	Plant hormone Signal transduction
GinfChr2G00029970.1	K14516	ERF1	map04075	Plant hormone Signal transduction
GinfChr1G00101730.1	K14516	ERF1	map04075	Plant hormone Signal transduction
GinfChr1G00100110.1	K14516	ERF1	map04075	Plant hormone Signal transduction
GinfChr6G00239400.1	K13422	MYC2	map04016	Plant hormone Signal transduction
GinfChr6G00267160.1	K13464	JAZ	map04075	Plant hormone Signal transduction
GinfChr3G00137170.1	K13464	JAZ	map04075	Plant hormone Signal transduction
GinfChr5G00168240.1	K13464	JAZ	map04075	Plant hormone Signal transduction
GinfChr6G00248400.1	K13464	JAZ	map04075	Plant hormone Signal transduction
GinfChr6G00266780.1	K13464	JAZ	map04075	Plant hormone Signal transduction
GinfChr1G00095650.1	K12126	PIF3	Map04712	Plant hormone Signal transduction
GinfChr2G00027290.1	K13495	CISZOG	map00908	Zeatin biosynthesis
GinfChr6G00255370.1	K00588	E2.1.1.104	map00941	Flavonoid biosynthesis
GinfChr4G00071880.1	K00660	CHS	map00941	Flavonoid biosynthesis
GinfChr4G00071840.1	K00660	CHS	map00941	Flavonoid biosynthesis
GinfChr4G00071870.1	K00660	CHS	map00941	Flavonoid biosynthesis
GinfChr3G00137770.1	K00660	CHS	map00941	Flavonoid biosynthesis
GinfChr3G00124890.1	K22845	PGT1	map00941	Flavonoid biosynthesis
GinfChr5G00197280.1	K08243	CHR	map00941	Flavonoid biosynthesis
GinfChr1G00085290.1	K00487	CYP73A	map00941	Flavonoid biosynthesis
GinfChr5G00197500.1	K10775	PAL	Map00940	Phenylpropanoid biosynthesis
GinfChr1G00091980.1	K10775	PAL	Map00940	Phenylpropanoid biosynthesis
GinfChr1G00120940.1	K10775	PAL	Map00940	Phenylpropanoid biosynthesis
GinfChr2G00031750.1	K10775	PAL	Map00940	Phenylpropanoid biosynthesis
GinfChr2G00031750.1	K10775	PAL	Map00940	Phenylpropanoid biosynthesis
GinfChr5G00165360.1	K01904	4CL	Map00940	Phenylpropanoid biosynthesis
GinfChr4G00082360.1	K01904	4CL	Map00940	Phenylpropanoid biosynthesis

### 3.9 Gene expression and metabolite accumulation in flavonoid biosynthesis

**Figure 10:**
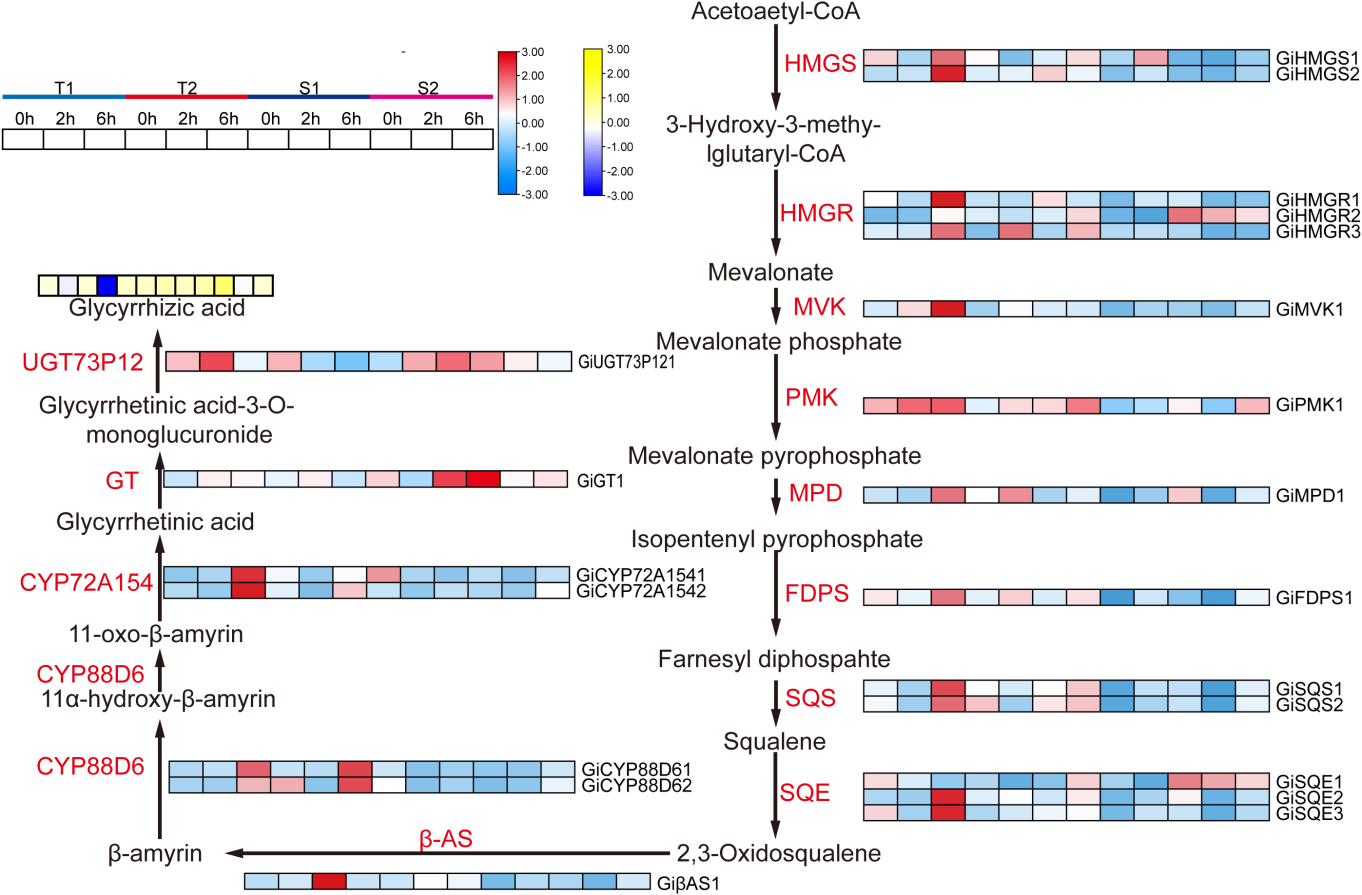
The synthesis pathway of glycyrrhizic acid and the expression status of related genes as well as the changes in glycyrrhizic acid content. HMGS, 3 - Hydroxy - 3 - methylglutaryl Coenzyme A synthase; HMGR, 3 - Hydroxy - 3 - methylglutaryl Coenzyme A reductase; MVK, Mevalonate kinase; PMK, Phosphomevalonate kinase; MPD, Mevalonate diphosphate decarboxylase; FDPS, Farnesyl diphosphate synthase; SQS, Squalene synthase; SQE, Squalene epoxidase; β-AS:β - Amyrin synthase; CYP, Cytochrome P450 monooxygenase; GT, Glycosyltransferase.

Based on WGCNA and transcriptome results, we screened genes associated with active ingredient synthesis from the target modules and enrichment analysis. The accumulation levels of major active ingredients across different time points are presented in **Figures 10** and **6**. As shown in **Figure 10**, in the T1 *Glycyrrhiza inflata* germplasm, 18 genes within the glycyrrhizin biosynthetic pathway (e.g., Gi*HMGS1*, Gi*HMGS2*, Gi*HMGR1*, Gi*HMGR2*) exhibited significant upregulation 6 hours post-stress. Combined with the accumulation results of active compounds across different stress durations, this pronounced upregulation likely contributed to T1’s substantially higher glycyrrhizin accumulation at 12 hours compared to other germplasms. While the T2 germplasm also showed upregulation of most genes in this pathway, the magnitude of expression change was less pronounced than in T1. In contrast, the stress-sensitive germplasms S1 and S2 reached peak expression levels for most glycyrrhizin pathway genes at 2 hours post-stress, followed by a decline at 6 hours. This expression pattern corresponds to their lower glycyrrhizin accumulation at 6 and 12 hours relative to T1 and T2.Conversely, within the Phenylpropanoid Metabolism Pathway (PMP), the T1 germplasm showed significant downregulation of gene expression at 2 and 6 hours post-stress (**Figure 6**), exhibiting an expression pattern opposite to that observed in the glycyrrhizin pathway. However, its constitutive (control) expression levels for PMP genes were notably higher than those in the other three germplasms. This elevated baseline likely explains T1’s sustained higher levels of flavonoid and isoflavonoid active ingredients during short-term stress, consistent with previous findings. Germplasms T2, S1, and S2 exhibited upregulation of PMP pathway genes at 2 and 6 hours post-stress, suggesting resource allocation towards synthesizing active compounds like Liquiritin, Licochalcone A, and Glabridin. Notably, S1 displayed higher expression of Gi*CHI1*, Gi*CHI4*, Gi*IFS1*, and Gi*GT1* at 6 hours compared to the other germplasms, resulting in significantly greater Glabridin synthesis post-stress. T2, meanwhile, upregulated genes such as Gi*CHS3*, Gi*CHS4*, Gi*CHS5*, Gi*CHR1*, and Gi*CHR2*, potentially facilitating increased Isoliquiritigenin and subsequent Liquiritin production.

## 4 Discussion

### 4.1 Analysis of quality traits and salt-alkali stress tolerance in *Glycyrrhiza inflata* germplasm resources from Tarim basin

This study revealed that all 29 tested accessions of *Glycyrrhiza inflata* exhibited germination rates exceeding 45% under saline-alkaline stress (pH 8.15), with a mean germination rate of 65.13%. Notably, several accessions achieved rates surpassing 80%. Plant height ranged from 1.74 m to 3.24 m, collectively demonstrating the inherent capability of these diverse *G. inflata* germplasms from the Tarim Basin periphery to thrive in desert saline-alkali soils. Furthermore, the accessions displayed substantial variation in key bioactive constituents: total flavonoid content ranged from 17.926 ± 2.810 mg/g to 28.966 ± 5.533 mg/g, glycyrrhizic acid content from 2.056 ± 0.231 mg/g to 7.506 ± 1.480 mg/g, and liquiritin content from 0.717 ± 0.139 mg/g to 4.008 ± 1.232 mg/g. These results, particularly the high levels of the primary quality indicators glycyrrhizic acid and liquiritin, highlight.

### 4.2 Changes in key bioactive compounds, endogenous hormones, and antioxidant enzyme activities in *Glycyrrhiza inflata*

The primary bioactive constituents of *Glycyrrhiza inflata* are triterpenoids and flavonoids (Yang et al., 2017), These compounds not only exhibit free radical scavenging capacity and antioxidant activity (Kumar and Pandey, 2013), but also function as plant growth regulators (Kumar and Pandey, 2013), playing crucial roles in plant adaptation to environmental conditions and defense against abiotic stress (Shen et al., 2022), Furthermore, studies in both model (Ma et al., 2019) and non-model organisms (Zhang et al., 2023) have demonstrated that appropriate salt stress can effectively enhance desirable traits in cultivated germplasm. Consequently, we subjected roots of four *G. inflata* germplasms to hydroponic treatment with 150 mM NaCl (the previously determined optimal concentration in our laboratory) for 0h,2h, 6h. Subsequently, we measured changes in physiological parameters, endogenous hormone levels, and key bioactive compound concentrations. Following salt stress exposure, all four germplasms exhibited a characteristic triphasic response in CAT, SOD, and POD enzyme activities: an initial increase, followed by a decline, and then a secondary rise, ultimately stabilizing at levels slightly above or comparable to the control group (**Figure 3**). Generally, the salt-tolerant germplasms (T1, T2) displayed higher POD, and CAT activities and lower MDA content across most time points compared to the salt-sensitive germplasms (S1, S2). Chlorophyll and free proline content showed no significant changes post-stress, consistent with experimental expectations. This dynamic pattern aligns with findings reported by Ma et al. (Ma et al., 2025b)and Zhang et al(Zhang et al., 2023 **).**Concurrently, endogenous hormone levels in all four germplasms underwent varying degrees of fluctuation, representing a physiological effort to mitigate the detrimental effects of salt stress on plant growth. Changes in abscisic acid (ABA), indole-3-acetic acid (IAA), and zeatin (ZT) levels are shown in **Figures 4A–C**. Notably, S1 displayed a significant decrease in ABA post-stress contrary to its established role as a key stress-responsive hormone in plants (Baron et al., 2012) which may contribute to its lower stress resistance. Salt stress typically triggers resource reallocation from growth to defense mechanisms, reducing auxin (IAA) demand and synthesis (Aizaz et al., 2024). Consistent with this, tolerant germplasms T1 and T2 showed significant IAA downregulation at 2h and 6h post-stress. In contrast, sensitive types S1 and S2 exhibited initial IAA increases; this delayed or attenuated stress response likely contributes to their reduced tolerance. Similarly, ZT levels in T1 remained stable throughout the 6h stress period, mirroring its ABA and CAT response patterns. T2, S1, and S2 displayed significant ZT increases at 2h followed by declines at 6h, representing an adaptive effort to mitigate stress impact. Numerous studies confirm that plants enhance flavonoid synthesis under salt stress to counteract its effects (Xu et al., 2020; Feng et al., 2023), establishing controlled salt exposure as an effective strategy for boosting bioactive compound production. As shown in **Figure 5**, all four germplasms exhibited significant upregulation of key bioactive compounds post-stress. Intriguingly, under control conditions, one-year-old plants from all four germplasms—particularly T1, T2, and S2—showed no significant differences in major bioactive compound levels (**Figure 2**). However, root exposure to 150 mM NaCl induced distinct accumulation patterns: T1/T2 accumulated substantially higher Glycyrrhizic acid than S1/S2; S1/S2 accumulated substantially higher Glabridin and LicochalconeA than T1/T2; Integrating these findings with preceding results, we infer that differential stress-induced expression of genes within flavonoid and glycyrrhizin biosynthetic pathways across the four germplasms underlies these compound-specific accumulation patterns.

### 4.3 Salt stress-responsive gene expression

**Figure 11:**
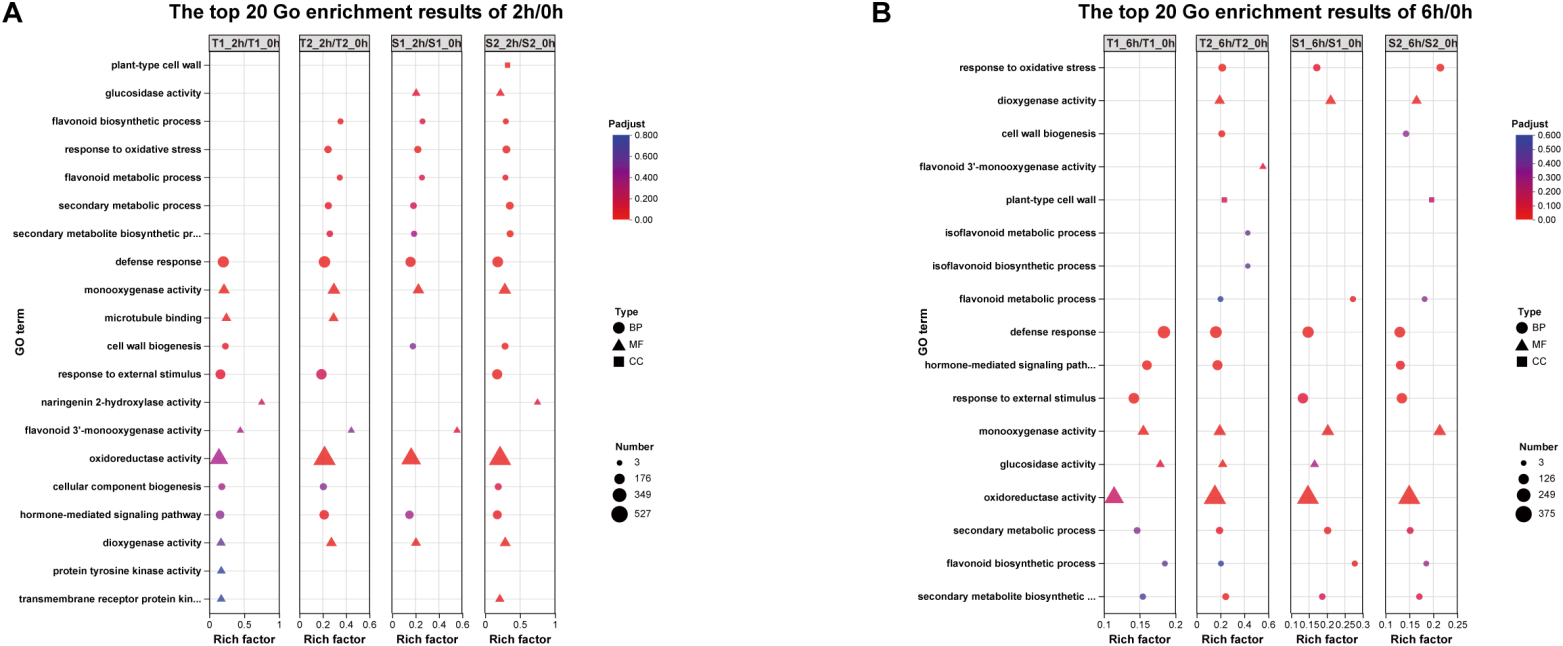
**(A)** The top 20 Gene ontology (GO) enrichment results of 2h/0h DEGs. **(B)** The top 20 Gene ontology (GO) enrichment results of 6h/0h DEGs.

**Figure 12:**
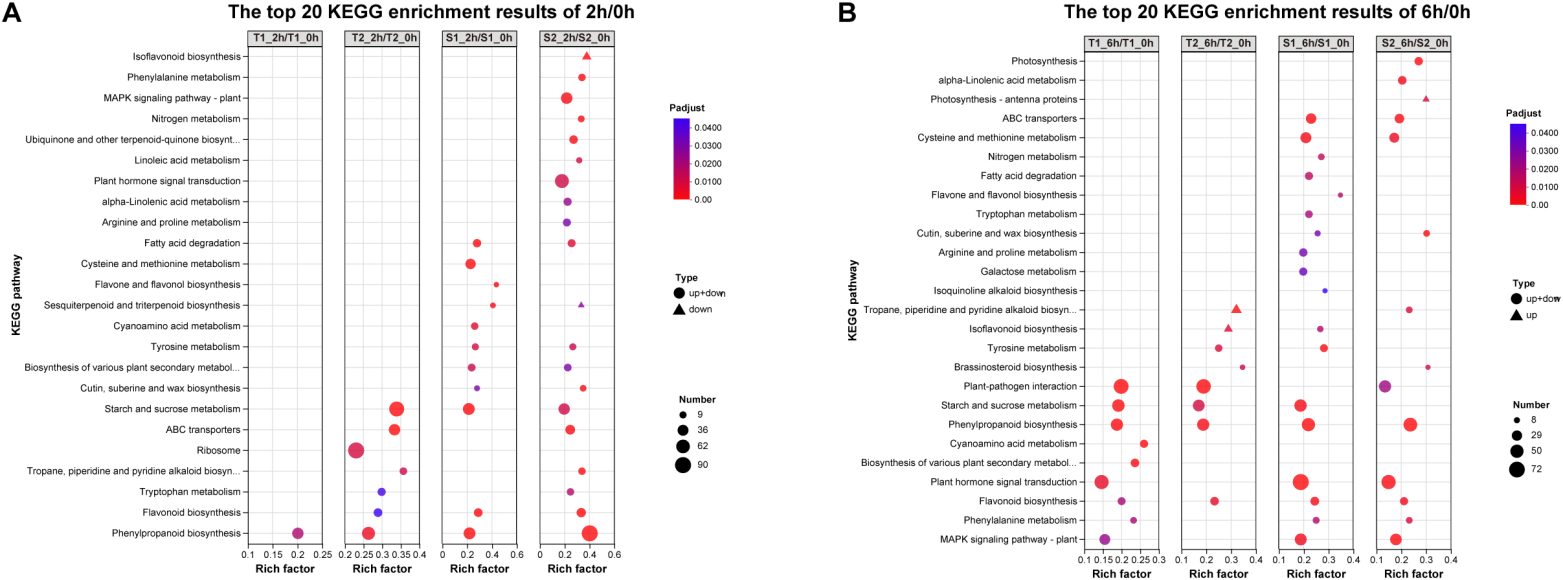
**(A)** The top 20 KEGG pathway enrichment results of 2h/0h DEGs. **(B)** The top 20 KEGG pathway enrichment results of 6h/0h DEGs.

Transcriptome analysis in this study revealed substantial variation in the number of differentially expressed genes (DEGs) across different treatment combinations (**Figure 7**). The T2_6h/T2_0h comparison exhibited the highest number of upregulated genes (3,093, representing 6.43% of expressed genes), alongside 2,567 downregulated genes (5.96%). Conversely, the S2_6h/S2_0h comparison showed relatively fewer DEGs. Overall, most treatment groups induced significant transcriptional reprogramming, characterized by substantial numbers of both upregulated and downregulated genes. This reflects the complexity and diversity of gene expression regulation in different *Glycyrrhiza inflata* germplasms as they adapt physiologically to varying environmental or experimental conditions. Notably, the number of DEGs at 2h post-stress was slightly higher than at 6h.Gene Ontology (GO) enrichment analysis (**Figure 11**) indicated that *G. inflata* orchestrates a multifaceted response to salt stress by differentially regulating genes involved in diverse functions. These include, but are not limited to, flavonoid biosynthesis, modulation of oxidoreductase activity, plant hormone signal transduction, and amino acid degradation. Consistent with the DEG count data, GO terms were enriched with a greater number of genes at 2h compared to 6h, suggesting a more extensive transcriptional response during the initial phase of stress acclimation. Kyoto Encyclopedia of Genes and Genomes (KEGG) pathway enrichment (**Figure 12**) further highlighted significant differential expression of genes within key metabolic pathways post-stress, notably flavonoid biosynthesis, flavone and flavonol biosynthesis, and phenylalanine metabolism. The number of differentially expressed genes within these pathways varied considerably among the germplasms, potentially contributing to the observed differences in bioactive compound accumulation.

### 4.4 Biosynthetic regulatory gene network for key bioactive compounds in *Glycyrrhiza inflata*

Recent years have witnessed growing research interest in the clinical efficacy (Liu et al., 2025) therapeutic applications (Liu et al., 2021), and cosmetic utilization (Jiang et al., 2015) of bioactive compounds derived from licorice (Glycyrrhiza spp.). However, the specific biosynthetic mechanisms governing key active constituents, such as flavonoids and glycyrrhizin, in *Glycyrrhiza inflata* remain incompletely elucidated (Zhao et al., 2023). Furthermore, cultivated licorice, the primary supply source, faces challenges of declining quality, which constrains the development of related industries. Advances in bioinformatics have positioned Weighted Gene Co-expression Network Analysis (WGCNA) as a powerful tool for identifying target genes and key transcription factors (TFs) from RNA-seq data (He et al., 2023).In this study, we employed transcriptome analysis coupled with WGCNA to identify genes associated with bioactive compound accumulation in *G. inflata* under salt stress and constructed a putative biosynthetic network. Intriguingly, we identified a module (Black) exhibiting a strong negative correlation with Glabridin accumulation. Surprisingly, this module encompassed not only TFs involved in plant hormone signaling pathways (e.g., *JAZ*, *ERF1*, *PIF3*) but also the glycyrrhizin pathway rate-limiting enzyme *HMGR* and numerous genes encoding enzymes in flavonoid biosynthesis (*PAL*, *4CL*, *CHS*, *CHR*).Glycyrrhizin biosynthesis primarily occurs via the mevalonate (MVA) pathway. Within this pathway, 3-hydroxy-3-methylglutaryl-CoA reductase (*HMGR*), a key rate-limiting enzyme, catalyzes the conversion of HMG-CoA to mevalonate (MVA), leading to the formation of isopentenyl pyrophosphate (*IPP)* and dimethylallyl pyrophosphate (*DMAPP*) (**Figure 10**). Conversely, other flavonoid bioactive compounds originate from phenylalanine. Sequential catalysis by phenylalanine ammonia-lyase (*PAL*), cinnamate 4-hydroxylase (*C4H*), and 4-coumarate-CoA ligase (*4CL*) yields coumaroyl-CoA. This precursor is then channeled through various chalcone synthases (*CHS*) to synthesize distinct compounds like Glabridin, Liquiritin, and Licochalcone A (**Figure 6**).Current understanding suggests no direct regulatory interplay between these two pathways. Therefore, our WGCNA finding of a strong negative correlation between *HMGR* (a key enzyme in glycyrrhizin synthesis) and Glabridin accumulation was unexpected. However, integrating this result with phenotypic data (**Figures 5A, B**) revealed a compelling pattern: the T1 germplasm exhibited the highest glycyrrhizic acid accumulation post-stress but the lowest Glabridin levels among the four germplasms. Conversely, the S1 germplasm accumulated the highest Glabridin but showed no significant change in glycyrrhizic acid within the first 12h post-stress. This inverse relationship led us to hypothesize a potential regulatory mechanism linking these seemingly independent pathways, with *HMGR* potentially playing a pivotal role. Supporting this hypothesis, transcriptomic data revealed that in the T1 germplasm, genes within the glycyrrhizin pathway (including *HMGR*) were highly upregulated at 6h post-stress, significantly exceeding levels in other germplasms. Conversely, several genes in the Glabridin pathway (*PAL*, *C4H*, *4CL*) were downregulated. This coordinated differential expression across multiple genes in both pathways provides corroborative evidence for potential cross-talk. Furthermore, the Glabridin-correlated Black module contained *CHR* genes, which catalyze the formation of isoliquiritigenin. Notably, isoliquiritigenin, Liquiritin, Glabridin, and Licochalcone A all share coumaroyl-CoA as a common precursor. Their divergence hinges on the specific *CHS* isoform involved: *CHS* enzymes producing naringenin chalcone lead to isoliquiritigenin and Liquiritin, while those producing chalcone lead to Glabridin and Licochalcone A. Consequently, we infer that specific *CHS* isoforms (e.g., Gi*CHS4*, Gi*CHS5*, Gi*CHS6*, which were highly negatively correlated with Glabridin) may preferentially channel coumaroyl-CoA towards alternative flavonoid branches, thereby influencing Glabridin flux. Additionally, the module contained annotated TFs such as *ERF1*, *JAZ*, and *MYC*. *ERF1*, established in Arabidopsis thaliana, integrates environmental signals to promote local auxin (IAA) accumulation (Li et al., 2021). Elevated IAA typically diverts resources away from stress-related compound synthesis. *JAZ* proteins act as repressors in jasmonate (JA) signaling; their high expression dampens JA responses (He et al., 2023). Given that JA signaling enhances flavonoid accumulation in plants like tomato (Li et al., 2021), the presence of these TFs within the network further validates the biological relevance of our WGCNA findings.

## 5 Conclusion

*Glycyrrhiza inflata* is a halophytic plant thriving in desert environments, valued for its diverse medicinal compounds. It offers dual benefits: remediating saline-alkaline soils while generating local economic value. Preliminary screening of 29 germplasms from the Tarim Basin periphery revealed significant variation in bioactive compound profiles and germination rates under saline-alkaline conditions. Consequently, we selected two salt-sensitive germplasms (S1, S2) and two stress-tolerant germplasms (T1, T2) for controlled salt-stress experiments. Key findings indicate that one-year-old plants from all four germplasms showed no significant differences in total flavonoids or five target bioactive compounds under optimal growth conditions. However, salt stress significantly enhanced the accumulation of all five bioactive compounds, with their content exhibiting a time-dependent increasing trend over the stress duration. Concurrently, short-term salt stress boosted antioxidant enzyme activities as an immediate defense response. All four germplasms underwent significant metabolic reprogramming, characterized by substantial alterations in transcriptomic profiles, flavonoid biosynthetic pathways, and phytohormone signaling cascades, ultimately leading to divergent stress tolerance phenotypes. This study not only advances our understanding of the molecular regulatory mechanisms governing flavonoid biosynthesis under salt stress but also provides crucial genetic insights for the targeted improvement of flavonoid production in saline environments. Furthermore, it establishes a foundation for identifying elite *G. inflata* germplasms with superior bioactive compound yields and enhanced stress resilience. Such germplasms hold significant promise for combating soil salinization in the southern Xinjiang deserts and other arid regions globally.

## Data Availability

The raw data of the G. uralensis genome sequence have been deposited at the DNA Data Bank of Japan (DDBJ) under the Bioproject ID PRJDB3943.

## Supplementary material

Supplementary Figure 1 Three-dimensional evaluation coordinate system diagram of the quality and stress resistance of *G. inflata*

Supplementary Figure 2 The germination rates of *G. inflata* from four different provenance under salt treatment. The differences between samples were determined by one-way analysis of variance (ANOVA), and the significance difference when P < 0.05 was calculated by the least significant difference (LSD) test.

Supplementary Figure 3 The qRT-PCR analysis of 4 genes during various stages of saline-alkali stress. Data are the mean ± standard error of the three biological experiments, standard errors are shown as error bars above the columns. The red column indicates qRT-PCR results and the blue column indicates FPKM data, and all Data represent root tissue samples only.

Supplementary Figure 4 TIC chromatogram of 7 standard.

Supplementary Table 1 The table of latitude and longitude information for the collection sites of 29 different varieties of G. inflata. All Data represent root tissue samples only.

Supplementary Table 2 Correspondence Table of Gene Names and Gene IDs.

Supplementary Table 3 Real-time fluorescent quantitative PCR primer list.

Supplementary Table 4 List of quantitative compounds and MRM parameters.
