# Supplementary material for: Salt stress enhances bioactive compound accumulation in Glycyrrhiza inflata: integrated transcriptomics and physiological analysis reveals germplasm-specific adaptation mechanisms
Source: Front Plant Sci. 2025 Sep 3;16:1658530. doi: 10.3389/fpls.2025.1658530 (PMC12444188; doi:10.3389/fpls.2025.1658530)
Supplement: Supplementary Figure 1 — Three-dimensional evaluation coordinate system diagram of the quality and stress resistance of G. inflata [file DataSheet1.pdf]

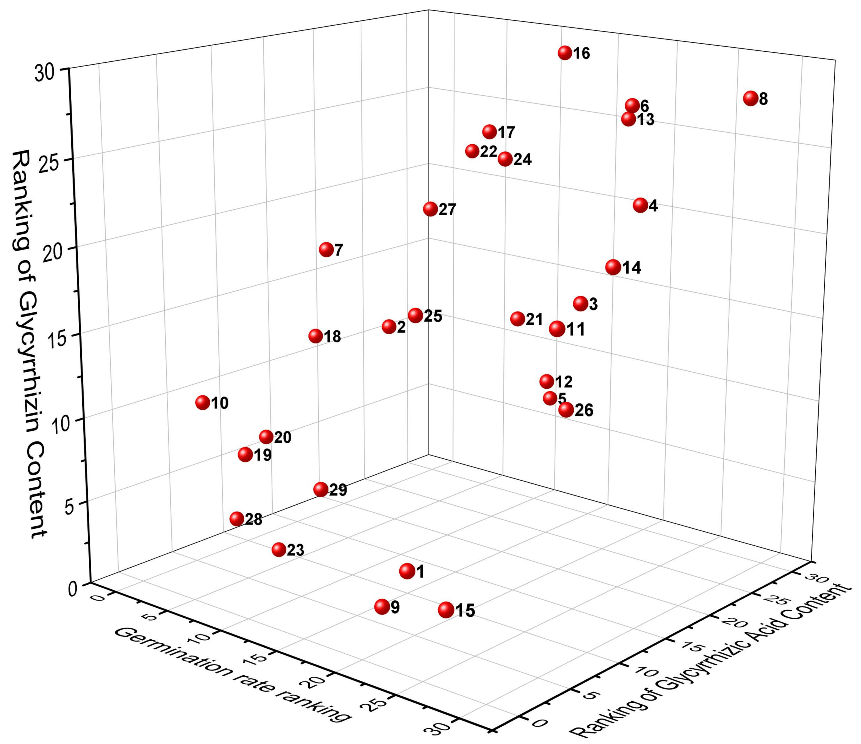

Supplementary Figure 1 | Three-dimensional evaluation coordinate system diagram of the quality and stress resistance of *G. inflata*
