# Supplementary material for: Salt stress enhances bioactive compound accumulation in Glycyrrhiza inflata: integrated transcriptomics and physiological analysis reveals germplasm-specific adaptation mechanisms
Source: Front Plant Sci. 2025 Sep 3;16:1658530. doi: 10.3389/fpls.2025.1658530 (PMC12444188; doi:10.3389/fpls.2025.1658530)
Supplement: Supplementary Figure 2 — The germination rates of G. inflata from four different provenance under salt treatment. The differences between samples were determined by one-way analysis of variance (ANOVA), and the significance difference when P < 0.05 was calculated by the least significant difference (LSD) test. [file DataSheet2.pdf]

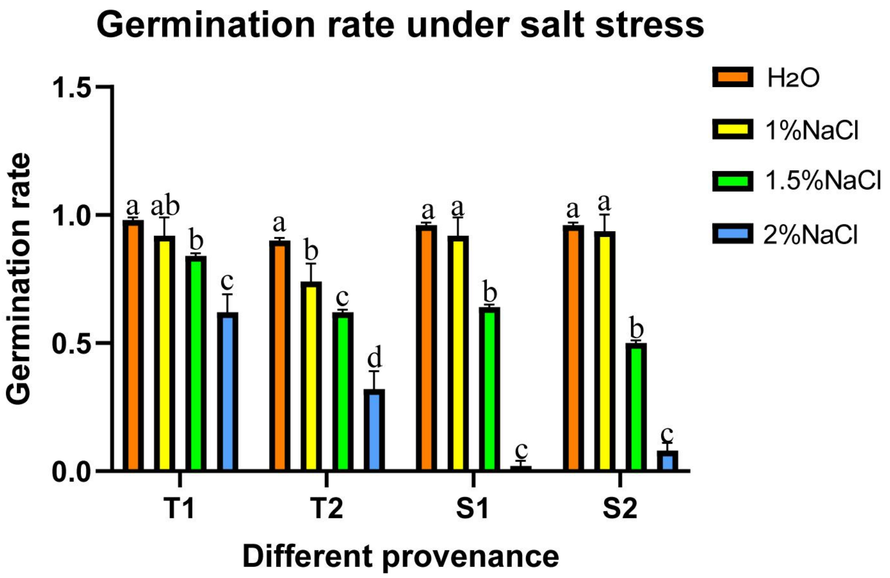

Supplementary Figure 2 | The germination rates of *G. inflata* from four different provenance under salt treatment.The differences between samples were determined by one-way analysis of variance (ANOVA), and the significance difference when $P < 0.05$ was calculated by the least significant difference (LSD) test.
