# Supplementary material for: Salt stress enhances bioactive compound accumulation in Glycyrrhiza inflata: integrated transcriptomics and physiological analysis reveals germplasm-specific adaptation mechanisms
Source: Front Plant Sci. 2025 Sep 3;16:1658530. doi: 10.3389/fpls.2025.1658530 (PMC12444188; doi:10.3389/fpls.2025.1658530)
Supplement: Supplementary Figure 3 — The qRT-PCR analysis of 4 genes during various stages of saline-alkali stress. Data are the mean ± standard error of the three biological experiments, standard errors are shown as error bars above the columns. The red column indicates qRT-PCR results and the blue column indicates FPKM data, and all Data represent root tissue samples only. [file DataSheet3.pdf]

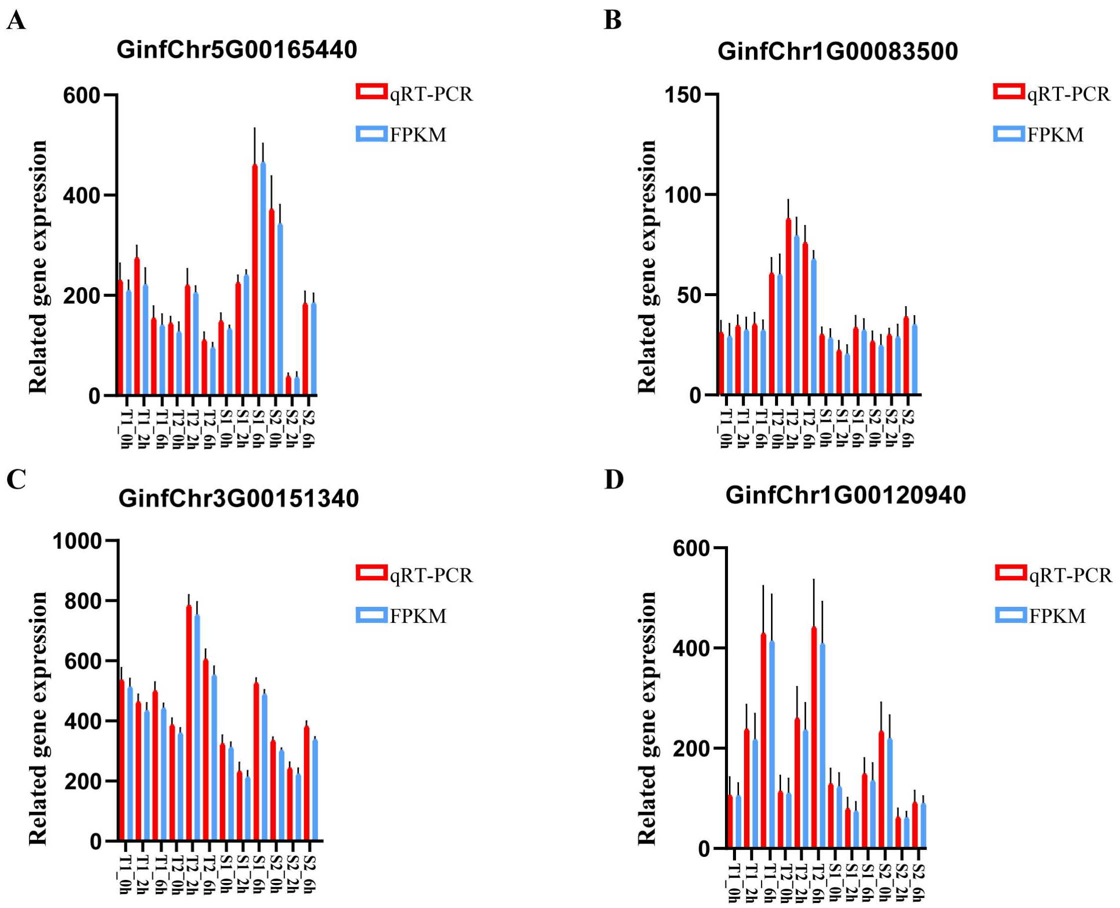

Supplementary Figure 3 | The qRT-PCR analysis of 4 genes during various stages of saline-alkali stress. Data are the mean ±standard error of the three biological experiments, standard errors are shown as error bars above the columns.The red column indicates qRT-PCR results and the blue column indicates FPKM data,and all Data represent root tissue samples only.
