# Supplementary figures and images for: Salt stress enhances bioactive compound accumulation in Glycyrrhiza inflata: integrated transcriptomics and physiological analysis reveals germplasm-specific adaptation mechanisms
Source: Front Plant Sci. 2025 Sep 3;16:1658530. doi: 10.3389/fpls.2025.1658530 (PMC12444188; doi:10.3389/fpls.2025.1658530)

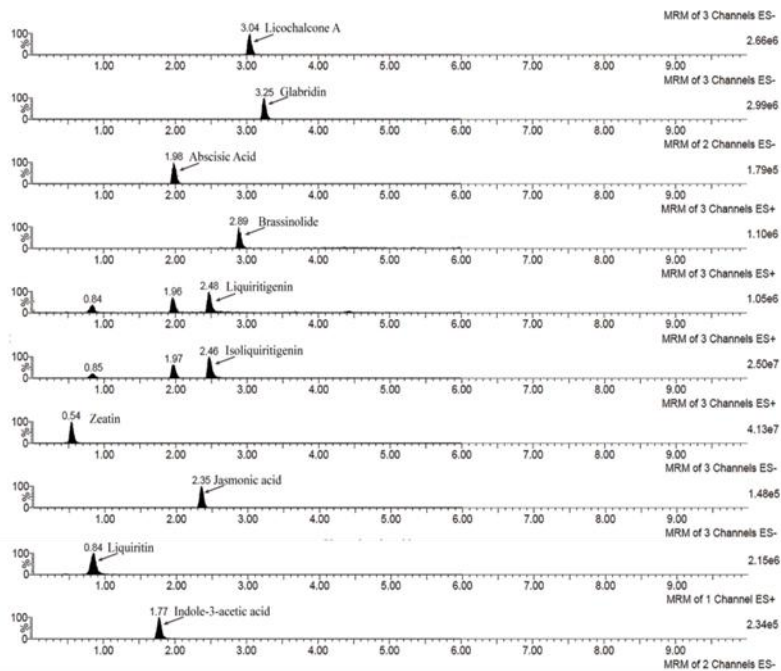

Supplement: Supplementary Figure 4 — TIC chromatogram of 7 standard. [file DataSheet4.pdf]
